# Harvest maturity is associated with carbon reallocation and fresh-to-flue-cured metabolic changes in *Nicotiana tabacum* leaves

**DOI:** 10.3389/fpls.2026.1939922

**Published:** 2026-09-21

**Authors:** Ao Gao, Chenyu Cai, Yuanfei Lou, Qi Xu, Kesu Wei, Changyun Zhang, Bo Zhang, Bohao Cheng, Xiaodong Xie, Shengjiang Wu, Gaoyi Cao

**Affiliations:** 1 College of Agronomy and Resources & Environment, Tianjin Agricultural University, Tianjin, China; 2 Guizhou Academy of Tobacco Science/Key Laboratory of Tobacco Quality and Ecology in Mountainous Areas, Guiyang, China; 3 Technology Center of China Tobacco Guizhou Industrial, Co., Ltd., Guiyang, China; 4 Zunyi Tobacco Company of Guizhou Province, Zunyi, China; 5 Zhangjiachuan Hui Autonomous County Agricultural Technology Extension Station, Tianshui, China

**Keywords:** carbon allocation, flue-curing, harvest maturity, *Nicotiana tabacum*, postharvest transformation, redox metabolism, untargeted metabolomics

## Abstract

Harvest maturity is associated with the biochemical status of leaf-harvested crops before postharvest processing, but the extent to which maturity-established metabolic differences are retained or transformed during flue-curing remains unclear in *Nicotiana tabacum*. Here, middle leaves of Yunyan 87 grown at a single experimental site were harvested at five harvest-maturity treatments (16, 18, 20, 22, and 24 days after topping). T1 and T5 were selected as contrasting harvest-maturity endpoints for paired metabolomic analyses of fresh leaves (F_T5 vs. F_T1), flue-cured leaves (FC_T5 vs. FC_T1), and direct fresh-to-flue-cured metabolite abundance changes at the same harvest-maturity treatment (FC_T1 vs. F_T1 and FC_T5 vs. F_T5). Phenotype, SPAD values, color traits, enzyme activities, conventional chemical constituents, auxiliary flue-cured leaf traits, and untargeted UPLC-HRMS metabolomics were integrated. Increasing maturity was accompanied by progressive yellowing, decreased SPAD and H° values, and increased L*, a*, b*, and C*. Fresh T5 leaves showed metabolic changes linked to carbon allocation, secondary metabolism, hormone/terpenoid-related pathways, phenylpropanoid-related metabolism, and redox adjustment. After flue-curing, maturity-associated differences were mainly reflected in aromatic amino acid metabolism, especially tryptophan and phenylalanine metabolism, together with secondary metabolism, metabolites assigned to transporter-associated KEGG categories, carbohydrate-related substrates, amino acid-related compounds, and lipid-associated remodeling. Direct fresh-to-flue-cured comparisons identified 84 and 85 differential metabolites at T1 and T5, respectively, including 70 shared metabolites and harvest-maturity-associated, comparison-specific components. These results identify a continuous physiological and conventional chemical maturity gradient across T1–T5 and endpoint metabolomic differences between the contrasting T1 and T5 treatments. Together, the findings suggest that harvest maturity is associated with both the fresh-leaf biochemical starting point and the metabolite abundance profile observed after flue-curing, although intermediate metabolomic states and causal mechanisms underlying individual metabolite changes remain to be determined.

## 1 Introduction

Tobacco (*Nicotiana tabacum* L.) is not only an important industrial crop but also a widely used model species for studies of plant development, specialized metabolism, and environmental responses (Li et al., 2024; Wu et al., 2020; Zhang et al., 2018). As a typical leaf-harvested plant, *N*. *tabacum* provides a useful system for investigating how leaf maturation coordinates pigment turnover, carbon allocation, redox regulation, and secondary metabolite biosynthesis. During the transition from active photosynthetic growth to maturation and senescence, leaves undergo extensive physiological and biochemical remodeling, including chlorophyll degradation, carbohydrate redistribution, changes in organic acid metabolism, and activation of specialized metabolic pathways (Guo et al., 2021; Havé et al., 2017). These processes determine the biochemical status of leaves before harvest and influence their subsequent biochemical changes during flue-curing, a postharvest dehydration and biochemical transformation process (Banožić et al., 2020; Chen et al., 2019a).

Leaf maturation is a complex developmental process rather than a simple external change in color or texture. Previous metabolomic studies have shown that tobacco leaves at different growth stages or stalk positions have distinct metabolic profiles, and several maturity-related compounds have been proposed as potential markers for ripeness assessment (Yang et al., 2025; Zhang et al., 2018). In addition to the developmental stage, regional ecological conditions can also reshape the metabolite composition of *N. tabacum* leaves, particularly metabolites associated with carbohydrate metabolism, nitrogen metabolism, lipid metabolism, and secondary metabolism (Sun et al., 2018). These findings indicate that the metabolic state of tobacco leaves is jointly shaped by developmental progression, environmental conditions, and flue-curing.

Carbon allocation is a central component of leaf maturation. As leaf maturity increases, chlorophyll content and photosynthetic activity gradually decline, while carbon skeletons are redistributed among soluble sugars, starch degradation products, respiratory intermediates, amino acids, and substrates for secondary metabolite biosynthesis. Such redistribution reflects a broader adjustment of plant carbon allocation and source–sink relationships during developmental progression (Hartmann et al., 2020). Such changes are particularly important for flue-cured leaves because sugars, amino acids, organic acids, and lipids participate in dehydration-associated biochemical reactions and contribute to the final metabolite pool (Liu et al., 2022; Hao et al., 2025; Wang et al., 2025). Similar maturity-dependent changes in metabolite accumulation and postharvest biochemical behavior have also been reported in other plant organs and harvested plant tissues (Farcuh et al., 2020; Turner et al., 2021). Therefore, maturity-dependent carbon reallocation provides an important entry point for understanding how leaf developmental status influences subsequent metabolic behavior in harvested plant tissues.

Secondary metabolism is another key feature of leaf developmental transition. Phenylpropanoid-related compounds, flavonoids, phenolic acids, terpenoids, alkaloids, polyamines, and lipid-derived metabolites participate in antioxidant regulation, cell-wall remodeling, defense responses, and the formation of metabolite precursors (Tang et al., 2020; Liu et al., 2022; Wang et al., 2025). In *N. tabacum* leaves, growth- and curing-associated metabolic changes are accompanied by shifts in phenylalanine metabolism, polyphenol metabolism, lipid degradation, and aroma-precursor-related pathways (Hao et al., 2025; Liu et al., 2022; Wang et al., 2025). However, how harvest-maturity-related metabolic differences established before harvest are reflected and remodeled after flue-curing remains insufficiently understood.

Recent studies have connected tobacco leaf characteristics with volatile composition, cell-wall components, plant -microbe interactions, and regional ecological differentiation (Cai et al., 2026; Jia et al., 2025; Zhong et al., 2025). In particular, Cai et al. (2026) reported maturity-associated metabolic differences in flue-cured tobacco from Dafang and Weining and linked cured-leaf metabolite profiles with regional style characteristics. That study clarified regional divergence in post-curing metabolic composition, but it did not directly resolve how metabolite profiles change from fresh leaves to flue-cured leaves at the same harvest maturity stage. Thus, an important unresolved question is whether harvest maturity mainly acts by establishing a distinct fresh leaf metabolic starting point before curing, by conditioning fresh-to-flue-cured metabolite abundance changes, or by both processes. Clarifying this issue requires paired fresh leaf and flue-cured leaf metabolomic comparisons under a single-site maturity gradient, rather than a regional style-comparison framework.

To address this gap, we used middle leaves of *Nicotiana tabacum* cultivar Yunyan 87 grown at one representative experimental site and established five harvest-maturity treatments corresponding to 16, 18, 20, 22, and 24 days after topping. This study differs from previous regional style-oriented work by focusing on within-site harvest maturity and paired fresh-to-flue-cured metabolomic comparisons. Four metabolomic comparisons were defined: F_T5 vs. F_T1 and FC_T5 vs. FC_T1 were used to identify harvest-maturity-associated endpoint differences within fresh leaves and within flue-cured leaves, respectively, whereas FC_T1 vs. F_T1 and FC_T5 vs. F_T5 were used to evaluate fresh-to-flue-cured metabolite abundance changes at the lower- and higher-maturity endpoints. In this study, fresh-to-flue-cured metabolic change was operationally defined as the net difference in relative metabolite abundance between fresh and flue-cured leaves harvested under the same harvest-maturity treatment and normalized on an equivalent dry-tissue-mass basis. This term does not imply that the observed abundance difference resulted from *de novo* biosynthesis or a single biochemical mechanism during flue-curing. By integrating fresh-leaf phenotype, SPAD values, color traits, key enzyme activities, conventional chemical components, auxiliary flue-cured leaf trait indices, and untargeted metabolomics, we aimed to: (i) characterize physiological and conventional chemical trends across the five harvest-maturity treatments; (ii) identify endpoint metabolomic differences between the contrasting T1 and T5 treatments in fresh and flue-cured leaves; (iii) evaluate fresh-to-flue-cured metabolite abundance changes at the two endpoint harvest-maturity treatments; and (iv) identify candidate pathway-level metabolite modules associated with these endpoint contrasts.

## 2 Materials and methods

### 2.1 Plant materials and experimental site

The field experiment was conducted during 2024–2025 at the Science and Technology Park in Liulong Town, Dafang County, Bijie City, Guizhou Province, China. The flue-cured tobacco cultivar *Nicotiana tabacum* L. cv. Yunyan 87 was used as the plant material, and seeds were provided by the Tobacco Breeding Center of Guizhou Academy of Tobacco Science. Yunyan 87 was selected because it is a widely cultivated flue-cured tobacco cultivar in Guizhou and southwestern China, has stable agronomic performance under local production conditions, and shows a clear maturity progression suitable for evaluating harvest-maturity-associated physiological and metabolomic differences. Tobacco seedlings were transplanted in mid-April with a row spacing of 120 cm and a plant spacing of 50 cm. Field management was performed according to the technical regulations for high-quality flue-cured tobacco production in Guizhou Province and the Code of Practice for Flue-Cured Tobacco Cultivation (GB/T 23221-2008). After topping, the 10th leaf counted from the bottom of the plant was used for sampling.

### 2.2 Experimental design and sample collection

Five harvest-maturity treatments were established according to the number of days after topping: T1, 16 days after topping; T2, 18 days after topping; T3, 20 days after topping; T4, 22 days after topping; and T5, 24 days after topping. These treatments were experimentally defined harvest-time points along a continuous maturation gradient rather than universally standardized maturity categories. The sampling window was selected with reference to local flue-cured tobacco production practice, the expected harvest period of middle-position leaves, and visible maturity-related traits, including leaf color transition, yellowing degree, leaf texture, and senescence symptoms. The experiment was conducted in a flat field with relatively uniform fertility and growth conditions. Fifteen plots were arranged in a randomized design, with three independent biological replicate plots for each harvest-maturity treatment, and each plot covered 0.089 ha.

Standard bulk-curing barns with downward airflow were used for flue-curing. In this study, flue-curing was regarded as the standard postharvest dehydration and biochemical transformation procedure for harvested tobacco leaves. The loading chamber measured 8.0 m × 2.8 m × 3.5 m. Leaves from different harvest-maturity treatments were tied to separate labeled rods and placed at adjacent positions on the middle tier of the same barn, 10–20 rods away from the barn door. Curing was conducted according to the technical regulations for tobacco leaf curing in Guizhou.

Fresh leaves from T1–T5 were sampled in the field to determine phenotypic traits, leaf color, SPAD values, and enzyme activities. Fresh samples used for enzyme activity and metabolomic analyses were immediately frozen in liquid nitrogen and stored at −80 °C until analysis. After flue-curing, representative flue-cured leaves were collected from each field plot for conventional chemical component determination and auxiliary flue-cured leaf trait assessment. For each treatment, 1.0 kg of representative flue-cured leaves was prepared for expert-based auxiliary assessment.

Fresh leaves and flue-cured leaves from T1 and T5 were selected for untargeted metabolomic analysis because these two treatments represented the most contrasting lower- and higher-maturity endpoints based on the full T1–T5 phenotypic, physiological, and biochemical assessment. In this study, F indicates fresh leaves collected before flue-curing, and FC indicates leaves obtained after the standard flue-curing procedure. Four metabolomic sample groups were therefore prepared: F_T1, F_T5, FC_T1, and FC_T5.

For metabolomic analysis, six independently collected composite biological samples were prepared for each group. Because each field plot was relatively large, two spatially separated sampling units were established within each plot. Each sampling unit consisted of representative leaves collected from different plants within the corresponding plot. After removal of the leaf tip, basal portion, and petiole, leaf blades from the same sampling unit were pooled and homogenized to generate one composite biological sample. Thus, each metabolomic group contained six composite biological samples nested within three independent field plots, resulting in 24 metabolomic samples in total. Individual leaves within each sampling unit were treated as subsamples and were not considered independent biological replicates.

### 2.3 Instruments and reagents

A SPAD-502 chlorophyll meter (Konica Minolta Sensing Inc., Japan) and a CR-10 chroma meter (Konica Minolta Sensing Inc., Japan) were used. Other major instruments included a refrigerated high-speed centrifuge (Centrifuge 5430, Eppendorf), a vortex mixer (QL-901, Qilinbeier Instrument Manufacturing Co., Ltd., China), an ultrapure water purification system (Milli-Q Integral, Millipore, USA), a refrigerated vacuum concentrator (Maxi Vacbeta, GENE COMPANY) and a tissue grinder (JXFSTPRP, Jingxin, Shanghai, China). Methanol (A454-4) and acetonitrile (A998-4) were LC-MS grade (Thermo Fisher Scientific, USA). Ammonium formate (17843-250G) was purchased from Honeywell Fluka (USA), formic acid (50144–50 mL) was purchased from DIMKA (USA), and water was provided by the ultrapure water system. The internal standards were d_3_-leucine, ^13^C_9_-phenylalanine, d_5_-tryptophan and ^13^C_3_-progesterone.

### 2.4 Measurement of color parameters and SPAD values

Fresh-leaf color parameters were measured using a CR-10 portable chroma meter (Konica Minolta Sensing Inc., Japan). Lightness (L*), redness-greenness (a*) and yellowness-blueness (b*) in the CIELAB color space were recorded, and chroma (C*) and hue angle (H°) were calculated as C* = √(a*^2^ + b*^2^) and H° = arctan(b*/a*), respectively. Measurements were taken at symmetric positions on both sides of the leaf, approximately 5 cm from the midrib. Three points were evenly measured on each half leaf, giving six points per leaf. Fifteen leaves were measured for each biological replicate, and the mean value of these leaves was used as one replicate value for statistical analysis (Hu et al., 2021). Overall color difference was calculated as ΔE_aβ_ = [(ΔL)^2^ + (Δa*)^2^ + (Δb*)^2^]^1/2^, where ΔL*, Δa*, and Δb* represent differences in the respective CIELAB coordinates relative to the T1 centroid. For T2–T5, ΔE*ab was calculated at the biological-replicate level relative to the mean L*, a*, and b* values of T1.

Relative chlorophyll content was measured using a SPAD-502 chlorophyll meter (Konica Minolta Sensing Inc., Japan). SPAD values were recorded for the leaf tip, middle and base. For each treatment, three biological replicates were used; ten leaves per plot and measurements taken from symmetrical positions on both sides of each leaf were treated as subsamples, and the mean SPAD value of each biological replicate was used for statistical analysis.

### 2.5 Determination of conventional chemical components

Flue-cured leaves from different maturity treatments were oven-dried at 60 °C, ground and passed through a 60-mesh sieve. Water-soluble total sugar, reducing sugar, total alkaloids, potassium and chloride were determined using an AUTOAnalyzer 3 continuous-flow analyzer (AA3, Bran+Luebbe, Germany), while total nitrogen was determined using a Futura II continuous-flow analyzer (AMS Alliance, France). Determination of water-soluble total sugar, reducing sugar, total alkaloids, total nitrogen and chloride followed the continuous-flow method (YC/T 159-2019), and potassium determination followed the continuous-flow method (YC/T 217-2007).

### 2.6 Expert-based auxiliary assessment of flue-cured leaf traits

Expert-based auxiliary assessment was used to characterize flue-cured leaf traits and was not used as the primary basis for interpreting maturity-associated metabolic regulation. The assessment was conducted in accordance with the tobacco industry standards YC/T 530–2015 and YC/T 138-1998. Seven trained tobacco-leaf assessors were organized by Guizhou Academy of Tobacco Science.

Before assessment, all flue-cured leaf samples were coded with random sample numbers, and the assessors were not informed of the corresponding maturity treatments. Each assessor scored the samples independently, without discussion during the scoring process. After scoring, the original scoring sheets were checked for completeness and obvious recording errors. The final value for each index was calculated as the mean score of the seven assessors. This procedure was used as a quality-control measure to reduce potential bias in the auxiliary flue-cured leaf trait assessment.

The full score for each individual index was 9 points. For strength, degree of fragrant style, and concentration of smoke, a higher score indicates greater intensity, whereas for the other indices a higher score indicates better performance. Concentration of smoke, degree of fragrant style, and strength were considered intensity-related auxiliary traits and were not included in the total score. Each assessor independently scored five indices, namely aroma quality, aroma quantity, offensive odor, irritation, and aftertaste. These indices were weighted using coefficients of 0.30, 0.30, 0.08, 0.15, and 0.17, respectively, to calculate the total auxiliary flue-cured leaf trait score as follows:

Total auxiliary flue-cured leaf trait score = (aroma quality × 0.30 + aroma quantity × 0.30 + offensive odor × 0.08 + irritation × 0.15 + aftertaste × 0.17) × 11.10.

### 2.7 Determination of key enzyme activities

Activities of chlorophyllase (CLH; ELISA kit, catalog no. ML-E-23878), polyphenol oxidase (PPO, catalog no. BC0195), peroxidase (POD, catalog no. BC0090), sucrose phosphate synthase (SPS, catalog no. BC0600), α-amylase (AMS, catalog no. BC0615), invertase (INV, catalog no. BC0130) and lipoxygenase (LOX, catalog no. BC0320) were determined. PPO activity was measured according to the kit instructions and with reference to the spectrophotometric principle reported for tobacco leaves (Broothaerts et al., 2000). Determination of POD, SPS and LOX was performed with reference to Henry et al. (1974); Huber and Huber (1991) and Waslidge and Hayes (1995), respectively, whereas CLH, AMS and INV activities were measured according to the manufacturers’ protocols. Each indicator was subjected to three biological replicates. The plant ELISA kit was purchased from Dongguan Mlbio Biotechnology Co., Ltd., and all other assay kits were purchased from Beijing Solarbio Science & Technology Co., Ltd.

Enzyme activity was expressed as U/mg protein. One unit (U) was defined as the amount of enzyme required to catalyze the conversion of 1 μmol of substrate per minute under the specified assay conditions, unless otherwise stated in the manufacturer’s instructions.

### 2.8 Metabolite extraction

Before extraction, fresh leaf samples were freeze-dried and ground to powder, and flue-cured leaf samples were also ground to powder. For both fresh and flue-cured leaf samples, 50 mg of powdered material was weighed into a 1.5 mL eppendorf tube for extraction. Thus, the direct fresh-vs.-flue-cured comparisons were performed on an equivalent dry-tissue-mass basis rather than on a fresh-weight basis. Peak areas were further normalized during data preprocessing and interpreted as relative metabolite abundance. Each sample was extracted with 800 μL of pre-chilled extraction solution (methanol/water = 7:3, v/v) together with 20 μL of internal standard solution. Samples were homogenized using a tissue grinder at 50 Hz for 10 min, followed by sonication in a 4 °C water bath for 30 min. After standing at −20 °C for 1 h, samples were centrifuged at 14,000 rpm and 4 °C for 15 min. The supernatant was filtered through a membrane and used for subsequent metabolomic analysis.

### 2.9 UPLC-MS analysis

Metabolites were separated and detected using a Waters 2777C UPLC system (Waters, USA) coupled to a Q Exactive HF high-resolution mass spectrometer (Thermo Fisher Scientific, USA). Separation was performed on a Hypersil GOLD aQ Dim column (1.9 μm, 2.1 × 100 mm; Thermo Fisher Scientific, USA). The mobile phases were water containing 0.1% formic acid as phase A and acetonitrile containing 0.1% formic acid as phase B. Elution was performed using the following gradient: 0–2 min, 5% B; 2–22 min, 5%–95% B; 22–27 min, 95% B; 27–27.1 min, 95%–5% B; and 27.1–30 min, 5% B. The flow rate was 0.3 mL/min, the column temperature was 40 °C, and the injection volume was 5 μL.

Full-scan MS and MS/MS data were acquired using the Q Exactive HF mass spectrometer (Thermo Fisher Scientific, USA). The m/z scan range was 125–1500 in positive ion mode and 100–1500 in negative ion mode. The full-scan resolution was 120,000; the AGC target was 1 × 10^6^ in positive mode and 3 × 10^6^ in negative mode; and the maximum injection time was 100 ms. According to precursor ion intensity, the top three ions were selected for fragmentation to obtain MS/MS information. The MS/MS resolution was 30,000; the AGC target was 2 × 10^5^ in positive mode and 1 × 10^5^ in negative mode; the maximum injection time was 50 ms; and the stepped normalized collision energies were set to 20, 40, and 60 eV. Electrospray ionization parameters were as follows: sheath gas flow rate, 40; auxiliary gas flow rate, 12; spray voltage, 3.80 kV in positive mode and 3.20 kV in negative mode; capillary temperature, 320 °C; and auxiliary gas heater temperature, 350 °C.

### 2.10 Metabolite identification, quality control, and data preprocessing

Raw mass spectrometry data were imported into Compound Discoverer 3.3 (Thermo Fisher Scientific, USA) and searched against BMDB (BGI Metabolome Database), mzCloud database and ChemSpider. The main annotation criteria were precursor-ion mass deviation < 5 ppm, fragment-ion mass deviation < 10 ppm, and retention-time deviation < 0.2 min. The resulting matrix contained metabolite peak areas and annotation information for downstream analysis.

Metabolite annotations were originally assigned using the BGI confidence-ranking system based on precursor-ion, MS/MS fragmentation, retention-time, and database-matching evidence. Because this vendor-specific ranking is not directly equivalent to the Metabolomics Standards Initiative (MSI) classification, conservative MSI confidence levels were additionally assigned according to the available analytical evidence. BGI Level 1 and Level 2 annotations supported by compound-specific MS/MS evidence were classified as MSI Level 2 putatively annotated compounds. BGI Level 3 annotations with lower-confidence spectral matches were classified as MSI Level 3 putative candidates. Features based only on precursor-ion or database matching were classified as MSI Level 3 when a candidate structure or compound class was available and as MSI Level 4 when no reliable structural information was available. Because authentic standards were not analyzed alongside the study samples, no metabolite was assigned MSI Level 1.

Pooled quality-control (QC) samples were prepared by combining aliquots from all samples and were injected at regular intervals throughout the analytical sequence. QC clustering in PCA space, internal-standard responses, and feature-level relative standard deviations were examined to evaluate instrumental stability and data preprocessing quality. Metabolite features with poor analytical reproducibility, defined as an RSD greater than 30% in pooled QC samples, were removed before downstream analysis. No procedural blank correction was applied because blank samples were not included in the analytical batch.

Peak areas were normalized before multivariate and univariate statistical analyses, and missing values were filtered or imputed according to the preprocessing workflow before downstream analysis. Metabolite annotations were interpreted conservatively, and pathway-level conclusions were based on coordinated metabolite groups rather than on single low-confidence features.

### 2.11 Statistical analysis

For physiological, color, enzyme-activity, and conventional chemical analyses, the field plot was used as the biological replicate, and subsample measurements within each plot were averaged before statistical testing. For metabolomic analyses, each independently collected composite sample was used as one biological sample for UPLC-HRMS profiling. Multivariate analysis, differential metabolite screening, and two-factor modeling were therefore performed using six composite biological samples per group. Because two composite samples were nested within each field plot, the nested sampling structure was explicitly described, and the results were interpreted as endpoint metabolite abundance patterns supported by replicated composite biological sampling rather than as evidence from six independent field plots.

Principal component analysis, hierarchical cluster analysis, and orthogonal partial least squares-discriminant analysis (OPLS-DA) were performed using identified metabolite peak areas as the input matrix. OPLS-DA model robustness was evaluated using seven-fold cross-validation and 200 permutation tests. Model parameters, including R^2^Y(cum), Q^2^(cum), and the R^2^ and Q^2^ intercepts from permutation testing, were reported. OPLS-DA was used only as an exploratory supervised visualization; biological interpretation was based primarily on PCA, QC-filtered differential metabolites, FDR-adjusted univariate statistics, pathway enrichment, and correlation analysis.

Differential metabolites were screened using the criteria VIP ≥ 1, FDR-adjusted Q < 0.05 and fold change ≥ 1.20 or ≤ 0.83. The Q value represents the false-discovery-rate-adjusted P value. For pathway enrichment analysis, only metabolites with identification levels 1–3 and non-NA pathway information were retained. Broad KEGG annotation categories were interpreted conservatively as database-derived pathway annotations rather than direct evidence of organism- or disease-specific biological processes.

Four metabolomic comparisons were considered. F_T5 vs. F_T1 and FC_T5 vs. FC_T1 were used to identify harvest-maturity-associated endpoint differences within fresh leaves and flue-cured leaves, respectively. FC_T1 vs. F_T1 and FC_T5 vs. F_T5 were used to evaluate fresh-to-flue-cured metabolite abundance changes at the lower- and higher-maturity endpoints. A two-factor analysis using harvest maturity, sample state, and their interaction was further used to distinguish main effects from maturity × sample-state interaction effects.

To characterize physiological and conventional chemical traits across the complete T1 -T5 harvest-maturity gradient, days after topping were treated as a continuous explanatory variable. Linear and quadratic regression analyses were performed for color parameters, ΔE*ab, SPAD values, and conventional chemical components using biological-replicate-level data. The linear model was specified as y = β0 + β1x + ϵ, and the quadratic model as y = β0 + β1xc + β2xc^2^ + ϵ, where x represents days after topping and xc represents centered days after topping. Linear-trend and quadratic-term P values were adjusted separately using the Benjamini–Hochberg false-discovery-rate procedure. The full regression outputs are provided in **Supplementary Table S3**. Data are presented as mean ± standard deviation. One-way ANOVA followed by Duncan’s multiple range test was performed in SPSS 26, and Pearson correlation analysis was used to examine associations between metabolites and traits. Figures were prepared using R, GraphPad Prism, Python, and an online bioinformatics visualization platform.

## 3 Results

### 3.1 Leaf maturation is associated with differences in phenotype, chlorophyll status, color traits, and chemical profiles after flue-curing

Fresh-leaf phenotype and the biochemical status of the corresponding flue-cured leaves differed markedly among harvest maturity levels (**Figures 1A, B**). With increasing maturity from T1 to T5, fresh leaves gradually changed from green to yellow-green and pale yellow. The main and lateral veins progressively lost greenness and turned whitish, while trichome shedding and maturity spots increased. These visual traits indicated continuous leaf maturation, with T5 showing the most advanced maturity phenotype among the five treatments.

**Figure 1:**
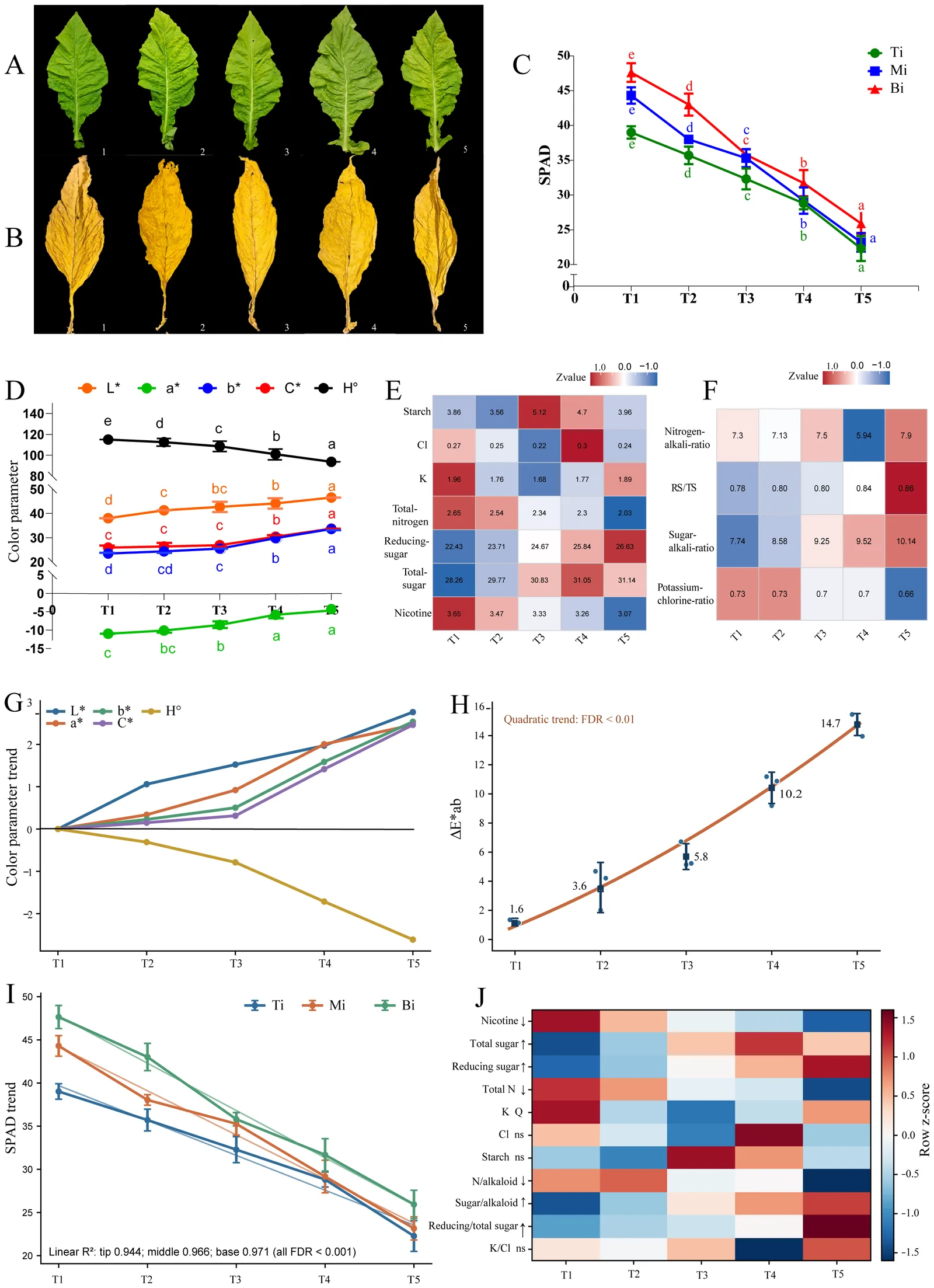
Phenotypic, physiological, and chemical changes during leaf maturation and flue-curing. **(A1–A5)** Phenotypes of fresh *Nicotiana tabacum* leaves from T1 to T5. **(B1–B5)** Phenotypes of the corresponding flue-cured leaves from T1 to T5. **(C)** SPAD values of fresh leaves at the leaf tip (Ti), middle (Mi), and base (Bi). **(D)** Color parameters of fresh leaves. **(E)** Z-score-standardized conventional chemical composition of flue-cured leaves. **(F)** Ratios of nitrogen/alkaloid, sugar/alkaloid, reducing sugar/total sugar, and potassium/chloride. **(G)** Standardized changes in L*, a*, b*, C*, and H° across the five harvest-maturity treatments. Values are expressed relative to T1. **(H)** Overall color difference relative to T1, expressed as ΔE*ab. Points and error bars represent mean ± SD of three independent biological replicates. The curve represents the fitted quadratic relationship with days after topping. **(I)** SPAD values measured at the leaf tip, middle, and base across the five harvest-maturity treatments. Points and error bars represent mean ± SD of three independent biological replicates, and lines represent fitted linear relationships with days after topping. **(J)** Z-score-standardized conventional chemical components across T1–T5.

SPAD values at the leaf tip, middle, and base decreased significantly with increasing maturity (P < 0.05; **Figure 1C**). By T5, SPAD values at the leaf tip and middle had both decreased to approximately 23.00. The positional differences observed at T1–T2, with leaf base > leaf middle > leaf tip, gradually converged from T3 onward, indicating improved within-leaf maturity uniformity at later harvest stages. Fresh-leaf color parameters also changed systematically with increasing harvest maturity (**Figure 1D**). Compared with T1, T5 leaves showed significant increases in L*, a*, b*, and C*, whereas the hue angle shifted toward yellow. These color changes were consistent with the decline in relative chlorophyll content reflected by SPAD values.

Linear and quadratic regression analyses based on biological-replicate-level data further supported a continuous but trait-specific physiological and conventional chemical maturity gradient across T1–T5 (**Figures 1G–J**; **Supplementary Table S3**). SPAD values at the leaf tip, middle, and base showed significant decreasing linear trends with days after topping, whereas ΔE*ab increased progressively from T2 to T5 and was best described by a nonlinear trend. Conventional chemical components also showed trait-specific trends: nicotine, total nitrogen, and the nitrogen/alkaloid ratio generally decreased, whereas total sugar, reducing sugar, the sugar/alkaloid ratio, and the reducing sugar/total sugar ratio generally increased. In contrast, potassium showed a nonlinear response, while chloride, starch, and the potassium/chloride ratio did not show stable monotonic trends. Thus, T2–T4 generally occupied intermediate positions for several major physiological and conventional chemical traits, although not all measured traits changed monotonically.

Relative to T1, ΔE*ab increased to approximately 3.6, 5.8, 10.2, and 14.7 at T2, T3, T4, and T5, respectively. The corresponding linear models for SPAD values at the leaf tip, middle, and base explained 94.4%, 96.6%, and 97.1% of the variation, respectively, and all three linear trends remained significant after FDR correction (FDR < 0.001).

The chemical composition of the corresponding flue-cured leaves also differed among harvest maturity treatments (**Figures 1E, F**). Total sugar and reducing sugar contents peaked at T5. The sugar-to-alkaloid ratio reached 10.14 at T5, and the reducing sugar-to-total sugar ratio reached 0.86. For mineral elements, T5 maintained a potassium content of 1.89% and a chloride content of 0.24%, resulting in a K/Cl ratio of 7.9. Auxiliary flue-cured leaf trait assessment further showed distinct postharvest trait profiles among maturity treatments (**Figure 2**). Together, the phenotypic, chemical, and auxiliary trait results showed that T1 and T5 represented clearly contrasting lower- and higher-maturity endpoints and were therefore selected for metabolomic comparison.

**Figure 2:**
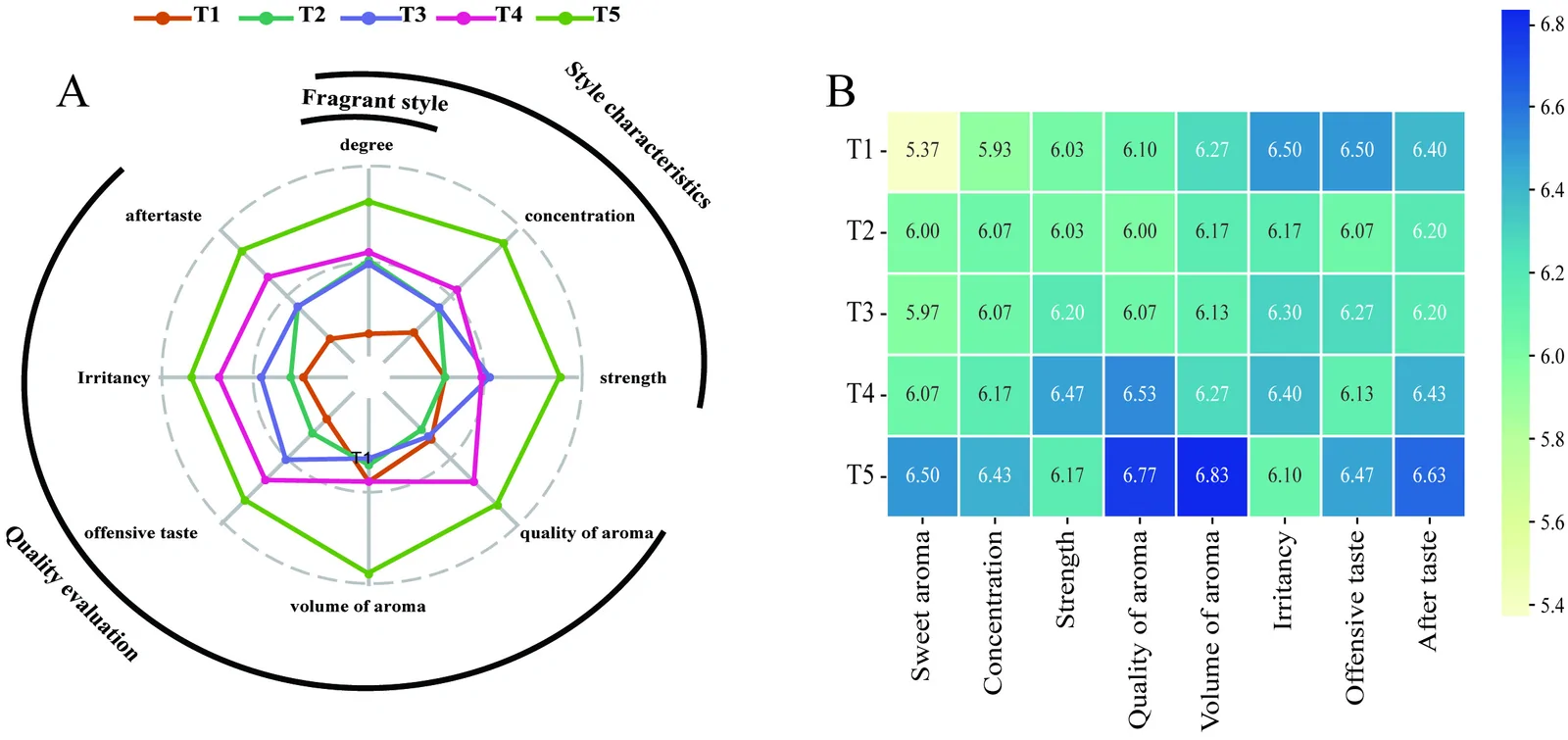
Auxiliary assessment of flue-cured leaves harvested at different maturity stages. **(A)** Z-score-standardized radar plot of auxiliary flue-cured leaf trait indices. **(B)** Heatmap of auxiliary flue-cured leaf trait indices. Concentration of smoke, degree of fragrant style, and strength were considered intensity-related auxiliary traits and were not included in the total auxiliary flue-cured leaf trait score.

### 3.2 Maturation was associated with differences in enzyme activities related to carbohydrate metabolism, pigment turnover, and redox status

To interpret the biochemical basis of maturity-related differences, key enzyme activities were compared between T1 and T5 fresh leaves (**Figure 3**). SPS and INV activities increased by 5.92% and 24.16%, respectively, in T5 leaves (P < 0.01), indicating maturity-associated differences in sucrose synthesis- and cleavage-related enzyme activities. AMS activity decreased slightly, whereas CLH activity showed no significant change. LOX activity increased significantly, PPO activity also increased, and POD activity decreased. These results showed coordinated maturity-associated differences in enzyme activities related to carbohydrate conversion, lipid oxidation, phenolic oxidation, and redox status.

**Figure 3:**
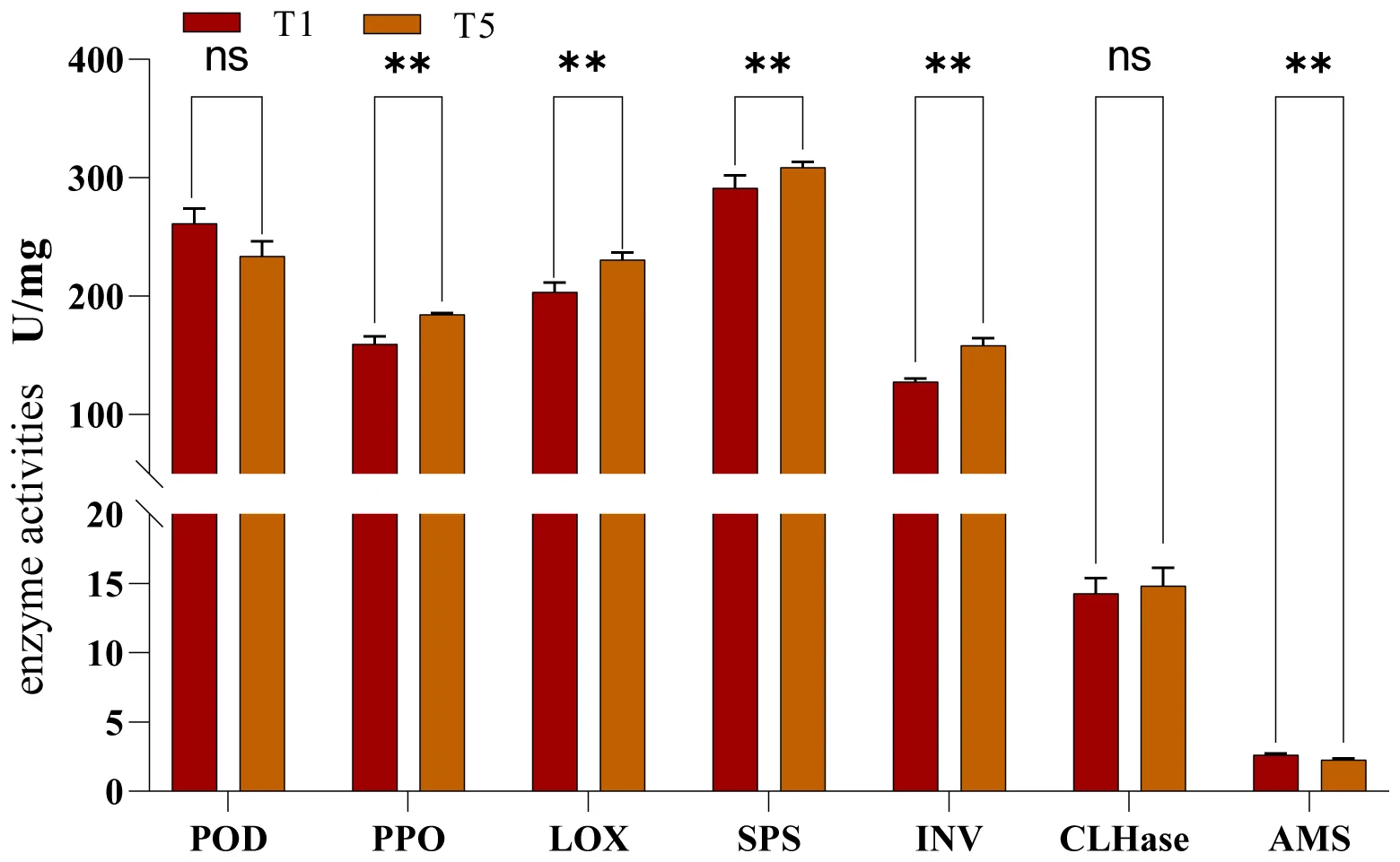
Key enzyme activities in fresh leaves at T1 and T5. Activities of enzymes related to pigment turnover, carbohydrate metabolism, lipid oxidation, phenolic oxidation, and redox regulation were compared between fresh leaves at lower- and higher- maturity endpoints. Data are shown as mean ± SD (n = 3 biological replicates). Double asterisks indicate significant differences between T1 and T5 at P < 0.01.

### 3.3 Untargeted metabolomics revealed distinct metabolic profiles between leaves at contrasting harvest-maturity endpoints

Untargeted metabolomic analysis was conducted on fresh and flue-cured leaves from T1 and T5 to compare maturity-associated metabolic differences before harvest and after flue-curing. A total of 2,140 metabolite features were annotated across F_T1, F_T5, FC_T1, and FC_T5 through overall metabolite profiling and database matching using BMDB, mzCloud, and ChemSpider. These annotated features included 359 terpenoids, 173 alkaloids, 153 flavonoids, 44 phenylpropanoids, 34 amino acid-related compounds, 27 lignans, 241 other compounds, 459 lipids, 129 benzenes and derivatives, 85 carbohydrates, 82 amino acids, peptides and analogues, 50 steroids and derivatives, 50 organic acids and 44 phenols and derivatives (**Supplementary Figure S1**; **Supplementary Table S1**).

Pooled QC samples formed a compact cluster in PCA space (**Supplementary Figure S2**), supporting the overall stability of the UPLC-HRMS analytical sequence. Internal standard responses were also examined to identify abnormal injections or obvious instrumental deviations. After QC-based filtering, including removal of features with high RSD values in pooled QC samples, the remaining metabolite features were used for multivariate analysis and differential metabolite screening.

PCA showed clear separation between F_T5 and F_T1, as well as between FC_T5 and FC_T1, indicating clear maturity-associated differences in metabolite composition in both fresh and flue-cured leaves (**Figures 4A, B**). OPLS-DA further supported the metabolic separation between lower- and higher-maturity samples.

**Figure 4:**
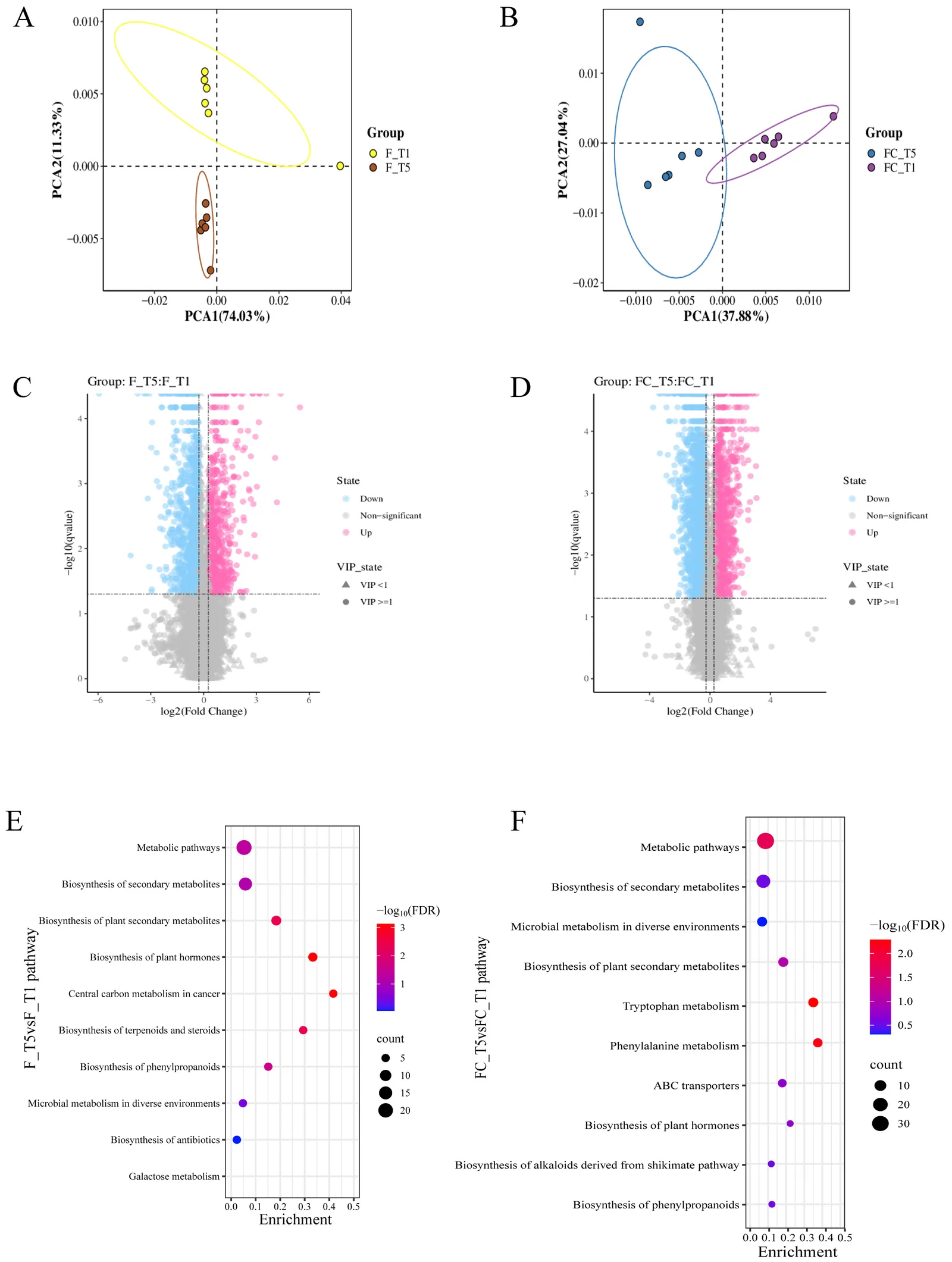
Multivariate analysis, differential metabolite screening, and KEGG pathway enrichment analysis. **(A)** PCA score plot of fresh leaves in the F_T5 vs. F_T1 comparison. **(B)** PCA score plot of flue-cured leaves in the FC_T5 vs. FC_T1 comparison. **(C)** Volcano plot of differential metabolites in fresh leaves. **(D)** Volcano plot of differential metabolites in flue-cured leaves. **(E)** Top enriched KEGG pathways of differential metabolites in fresh leaves. **(F)** Top enriched KEGG pathways of differential metabolites in flue-cured leaves. KEGG enrichment reflects database-derived pathway associations of annotated metabolites. Broad KEGG categories, such as microbial metabolism in diverse environments, were not interpreted as direct evidence of organism-specific or disease-related biological processes in tobacco leaves.

The OPLS-DA models showed high apparent classification performance, with R^2^Y(cum) = 0.999 and Q^2^(cum) = 0.941 for F_T5 vs. F_T1 and R^2^Y(cum) = 1.000 and Q^2^(cum) = 0.999 for FC_T5 vs. FC_T1. The corresponding Q^2^ intercepts from 200 permutation tests were −0.13 and −0.23, respectively (**Table 1**). Although these diagnostic results did not indicate obvious overfitting based on permutation testing, the OPLS-DA results were interpreted cautiously because supervised models can overestimate group separation in high-dimensional metabolomic datasets. Therefore, OPLS-DA was used only as exploratory supervised visualization, and biological interpretation was based primarily on PCA patterns, QC-filtered differential metabolites, FDR-adjusted univariate statistics, pathway enrichment, and correlation analysis.

**Table 1:** OPLS-DA model parameters for metabolomic comparisons between leaves at lower and higher-maturity endpoints.

Group	R^2^Y(cum)	Q^2^(cum)	R^2^ intercept	Q^2^ intercept
F_T5 vs F_T1	0.999	0.941	0.94	-0.13
FC_T5 vs FC_T1	1.000	0.999	0.90	-0.23

R^2^Y(cum) and Q^2^(cum) indicate the explanatory and predictive abilities of the OPLS-DA model, respectively. R^2^ and Q^2^ intercepts were obtained from 200 permutation tests. Model predictive ability was estimated using seven-fold cross-validation.

### 3.4 Differential metabolites indicated maturity-associated changes in carbon allocation, phenylpropanoid metabolism, and redox-related pathways

Differential metabolites were screened using the criteria VIP ≥ 1, FDR-adjusted Q < 0.05, and fold change ≥ 1.20 or ≤ 0.83. In the fresh-leaf comparison F_T5 vs. F_T1, 37 differential metabolites were identified. In the flue-cured leaf comparison FC_T5 vs. FC_T1, 59 differential metabolites were identified (**Figures 4C, D**; **Supplementary Table S1**).

In fresh leaves, KEGG enrichment analysis showed that differential metabolites in F_T5 vs. F_T1 were mainly associated with broad metabolic pathways and secondary metabolic processes, including metabolic pathways, biosynthesis of secondary metabolites, biosynthesis of plant secondary metabolites, biosynthesis of plant hormones, central carbon metabolism, biosynthesis of terpenoids and steroids, biosynthesis of phenylpropanoids, and galactose metabolism (**Figure 4E**; metabolite-level details are provided in **Supplementary Table S1**). Based on relative metabolite abundance, T5 fresh leaves showed increased accumulation of candidate metabolites associated with soluble carbohydrate metabolism, phenylpropanoid-related metabolism, polyamine metabolism, and redox adjustment, including sucrose, trans-cinnamaldehyde, syringin, spermidine, and oxidized glutathione. In contrast, several central-carbon-related metabolites, including citric acid, 2-oxoglutaric acid, and succinic acid, declined. These results suggest that higher maturity was associated with coordinated changes in carbon allocation, plant secondary metabolism, hormone/terpenoid-related pathways, and redox-related metabolites.

In flue-cured leaves, KEGG enrichment analysis showed that differential metabolites in FC_T5 vs. FC_T1 were mainly assigned to metabolic pathways, biosynthesis of secondary metabolites, biosynthesis of plant secondary metabolites, tryptophan metabolism, phenylalanine metabolism, ABC transporter-related categories, biosynthesis of plant hormones, biosynthesis of alkaloids derived from the shikimate pathway, and biosynthesis of phenylpropanoids (**Figure 4F**; metabolite-level details are provided in **Supplementary Table S1**). Among these terms, tryptophan metabolism and phenylalanine metabolism showed relatively strong enrichment signals, suggesting that aromatic amino-acid-related metabolite groups were important components of harvest-maturity-associated differences after flue-curing. Compared with FC_T1, FC_T5 showed lower relative abundance of several putatively annotated phenylalanine-derived and aromatic compounds, including phenylacetaldehyde and trans-cinnamic acid, together with changes in sugar-, amino acid-, lipid-, and transporter-associated substrate metabolites. Broad KEGG categories, such as microbial metabolism in diverse environments, were treated as database-derived annotation categories and were not interpreted as direct evidence of organism-specific biological processes in tobacco leaves. These results suggest that harvest-maturity-associated differences after flue-curing were mainly reflected in aromatic amino acid metabolism, secondary metabolism, transporter-associated substrate patterns, and carbohydrate-, amino acid-, and lipid-associated metabolite groups.

### 3.5 Direct fresh-to-flue-cured comparisons identify shared and harvest-maturity-associated metabolite abundance changes

To further characterize endpoint metabolite abundance differences between fresh and flue-cured leaves under different harvest-maturity backgrounds, direct comparisons between fresh and flue-cured leaves were performed at T1 and T5. The FC_T1 vs. F_T1 comparison identified 84 differential metabolites, including 57 metabolites with higher and 27 metabolites with lower relative abundance in flue-cured leaves relative to fresh leaves. The FC_T5 vs. F_T5 comparison identified 85 differential metabolites, including 61 with higher and 24 with lower relative abundance. Detailed metabolite information for these comparisons is provided in **Supplementary Table S1**. These results indicate extensive fresh-to-flue-cured metabolite abundance changes at both harvest-maturity endpoints, with comparable numbers of differential metabolites between the lower- and higher-maturity endpoints.

At the lower-maturity endpoint, the strongest increases after flue-curing included tentatively assigned naringin and neohesperidin, 3-methoxytyramine, 9-oxo-10(E),12(E)-octadecadienoic acid, L-aspartic acid, biotin, myricetin, and oleic acid, whereas uridine 5’-diphosphogalactose, 5’-S-methyl-5’-thioadenosine, beta-carotene, 2-oxoglutaric acid, oxidized glutathione, jasmonic acid, adenosine diphosphate, and L-leucine showed marked decreases. At the higher-maturity endpoint, representative metabolites with increased abundance after flue-curing included naringin, gibberellin A4, 3-methoxytyramine, 9-oxo-10(E),12(E)-octadecadienoic acid, oleic acid, L-kynurenine, and phenylacetaldehyde, whereas 5’-S-methyl-5’-thioadenosine, uridine 5’-diphosphogalactose, jasmonic acid, oxidized glutathione, beta-carotene, spermidine, 2-oxoglutaric acid, and L-leucine decreased. These abundance patterns suggested that fresh-to-flue-cured endpoint differences involved flavonoid-related compounds, lipid-derived metabolites, amino-acid-related substrates, nucleotide-related metabolites, carotenoid-related metabolites, and redox-associated compounds.

KEGG enrichment analysis further supported this interpretation. In FC_T1 vs. F_T1, 19 pathways were significantly enriched at FDR < 0.05, including biosynthesis of plant secondary metabolites, metabolic pathways, purine metabolism, fatty acid biosynthesis, phenylpropanoid biosynthesis, ABC transporters, flavonoid biosynthesis, biosynthesis of plant hormones, and tryptophan metabolism. In FC_T5 vs. F_T5, 22 pathways were significantly enriched, including metabolic pathways, biosynthesis of plant secondary metabolites, purine metabolism, protein digestion and absorption, tryptophan metabolism, ABC transporters, fatty acid biosynthesis, biosynthesis of plant hormones, biosynthesis of phenylpropanoids, and flavonoid biosynthesis. The repeated enrichment of plant secondary metabolism, phenylpropanoid/flavonoid-related pathways, fatty acid biosynthesis, purine metabolism, tryptophan metabolism, and ABC transporter-related categories suggested that these pathway-level metabolite groups were associated with fresh-to-flue-cured abundance differences. Broad KEGG categories, such as protein digestion and absorption, were considered database-derived annotations and were not interpreted as direct biological processes occurring in tobacco leaves. Therefore, these KEGG results were used to organize metabolite-level patterns rather than to infer specific pathway activity, transporter activity, or metabolic flux.

Venn analysis showed that FC_T1 vs. F_T1 and FC_T5 vs. F_T5 shared 70 differential metabolites, suggesting a broad shared set of fresh-to-flue-cured abundance changes. Only two metabolites, 9-oxo-10(E),12(E)-octadecadienoic acid and uridine 5’-diphosphogalactose, were shared by all four comparisons, suggesting that only a small subset of metabolites responded consistently across both maturity contrasts and fresh-to-flue-cured abundance changes. In addition, the FC_T1 vs. F_T1 comparison contained nine unique metabolites, including 2-hydroxycinnamic acid, chrysin, D-*alpha*-tocopherol, eucalyptol, geranylgeranyl pyrophosphate, and trans-aconitic acid, whereas FC_T5 vs. F_T5 contained six unique metabolites, including L-sorbose, protocatechuic acid, pyridoxal, and taxifolin. Together, these results identify a broad shared set of fresh-to-flue-cured metabolite abundance changes and a smaller set of harvest-maturity-associated, comparison-specific components.

To further distinguish harvest-maturity-associated and fresh-to-flue-cured abundance responses, a two-factor analysis was performed using harvest maturity, sample state, and their interaction as model terms. This analysis identified 902 metabolites with significant harvest-maturity main effects, 1,867 metabolites with significant sample-state main effects, and 698 metabolites with significant maturity × sample-state interaction effects at FDR < 0.05 (**Supplementary Figure S3**; **Supplementary Table S4**). The interaction term indicated that fresh-to-flue-cured abundance differences were not identical between T1 and T5 for a subset of metabolites. These results support harvest-maturity-dependent endpoint abundance differences but do not establish the biochemical origin of individual metabolite changes.

To place these direct fresh-to-flue-cured changes in the context of the maturity-associated pathway modules identified above, representative differential metabolites from the fresh- and flue-cured-leaf maturity contrasts were organized in a KEGG-style pathway map (**Figure 5**).

**Figure 5:**
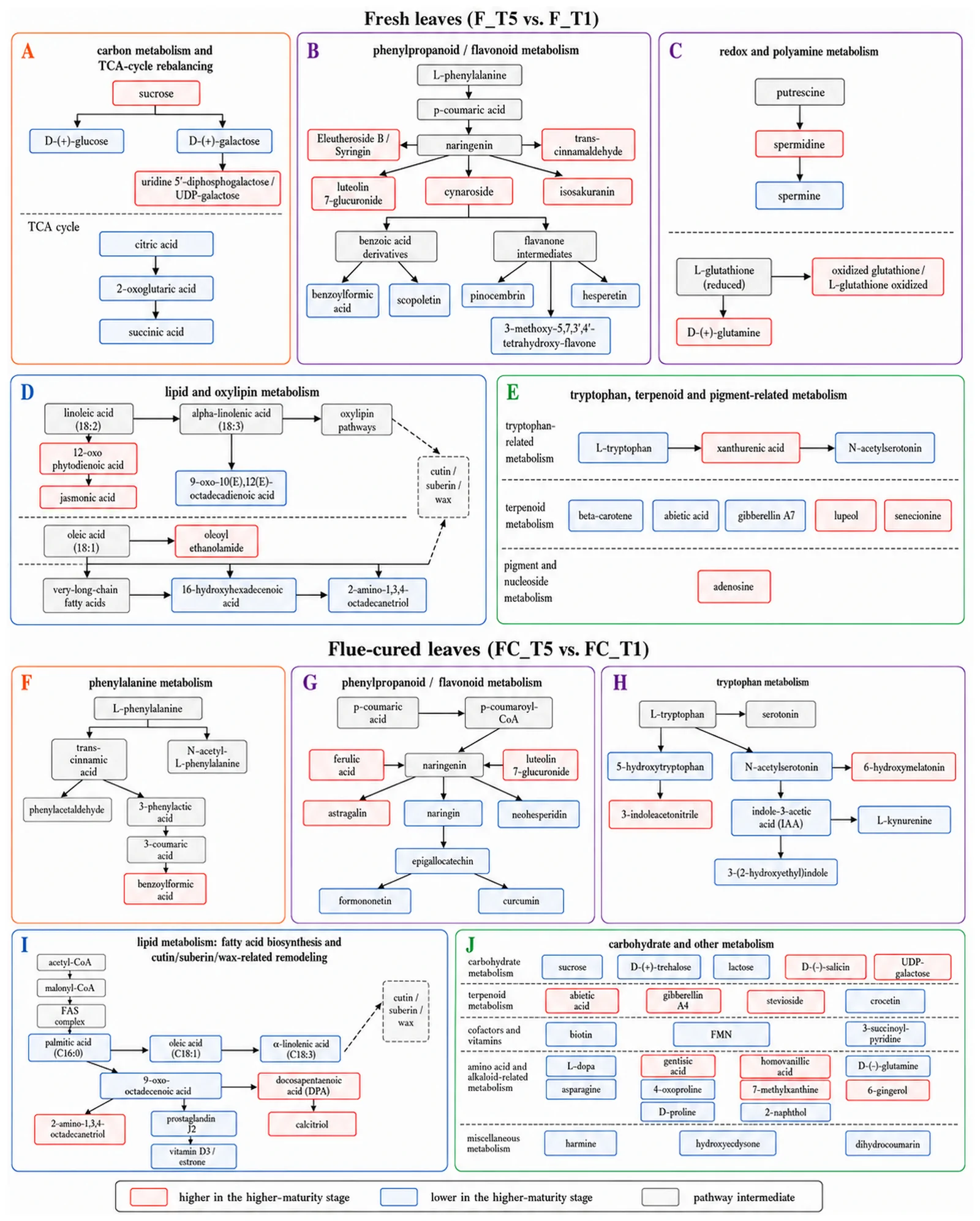
Representative pathway-level organization of putatively annotated maturity-associated metabolites in fresh and flue-cured leaves. **(A–E)** Fresh-leaf pathway modules in the F_T5 vs. F_T1 comparison, including central-carbon-related metabolism, phenylpropanoid/flavonoid metabolism, redox- and polyamine-associated metabolites, lipid and oxylipin metabolism, and tryptophan-, terpenoid-, and pigment-related metabolism. **(F–J)** Flue-cured leaf pathway modules in the FC_T5 vs. FC_T1 comparison, including phenylalanine metabolism, phenylpropanoid/flavonoid metabolism, tryptophan metabolism, lipid-associated remodeling, and carbohydrate, cofactor, vitamin, amino-acid and alkaloid-related metabolism. Red boxes indicate metabolites with higher relative abundance in the higher-maturity endpoint, blue boxes indicate lower relative abundance, and grey boxes indicate contextual pathway intermediates and do not necessarily represent significantly detected differential metabolites. This diagram summarizes pathway-level associations among putatively annotated metabolites and should not be interpreted as quantitative evidence of metabolic flux, enzyme activity, or causal pathway regulation.

To connect the direct fresh-to-flue-cured comparisons with the maturity-associated metabolite patterns identified above, hierarchical clustering was further used to visualize the abundance patterns of differential metabolites in the two maturity contrasts, F_T5 vs. F_T1 and FC_T5 vs. FC_T1 (**Figure 6**). In fresh leaves, metabolites associated with phenylpropanoid metabolism, polyamine metabolism, redox regulation, and selected sugar-related pathways showed clear maturity-dependent differences. In flue-cured leaves, the clustering pattern highlighted the coordinated changes in phenylalanine-derived metabolites, fatty acids, sugars, amino-acid-related compounds, and other substrates involved in flue-curing-based biochemical transformation.

**Figure 6:**
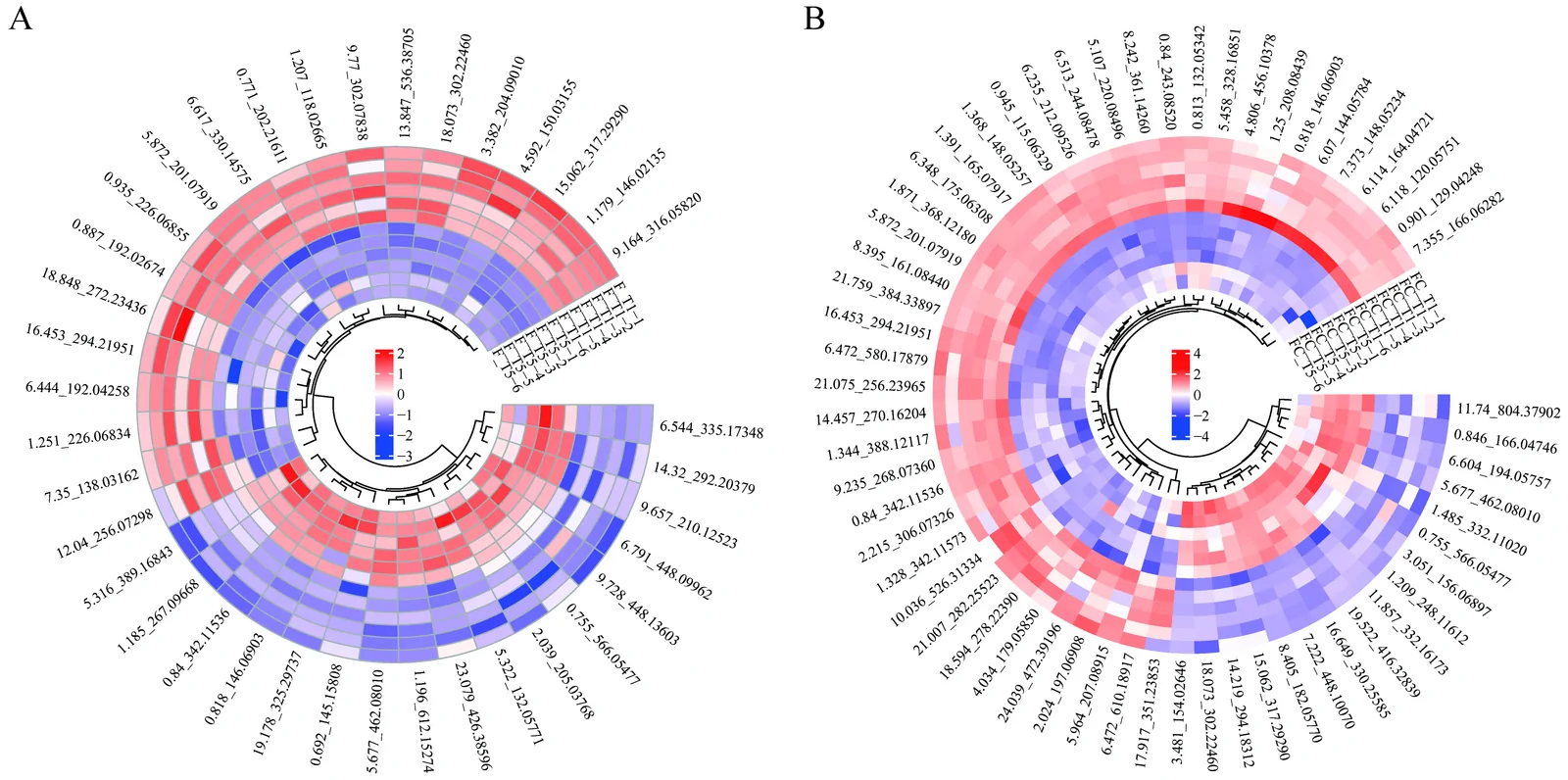
Cluster heatmap of differential metabolites in fresh and flue-cured leaves. **(A)** Cluster heatmap of differential metabolites in fresh leaves in the F_T5 vs. F_T1 comparison. **(B)** Cluster heatmap of differential metabolites in flue-cured leaves in the FC_T5 vs. FC_T1 comparison. The numbers on the side indicate metabolite identifiers corresponding to **Supplementary Table S2**.

### 3.6 Correlation analysis linked candidate metabolites with maturity-related physiological and biochemical traits

Correlation analysis was performed to identify candidate metabolites associated with physiological traits in fresh leaves and biochemical properties in flue-cured leaves (**Figure 7**). In fresh leaves, sucrose, citric acid, and succinic acid were mainly positively correlated with SPAD and/or color-related parameters, whereas oxidized glutathione showed an opposite trend. Spermidine was also associated with maturity-related physiological traits and oxidative enzyme activities. These correlation patterns suggest that candidate metabolites related to soluble carbon, organic acid metabolism, polyamine metabolism, and redox balance were linked with leaf maturity status before harvest.

**Figure 7:**
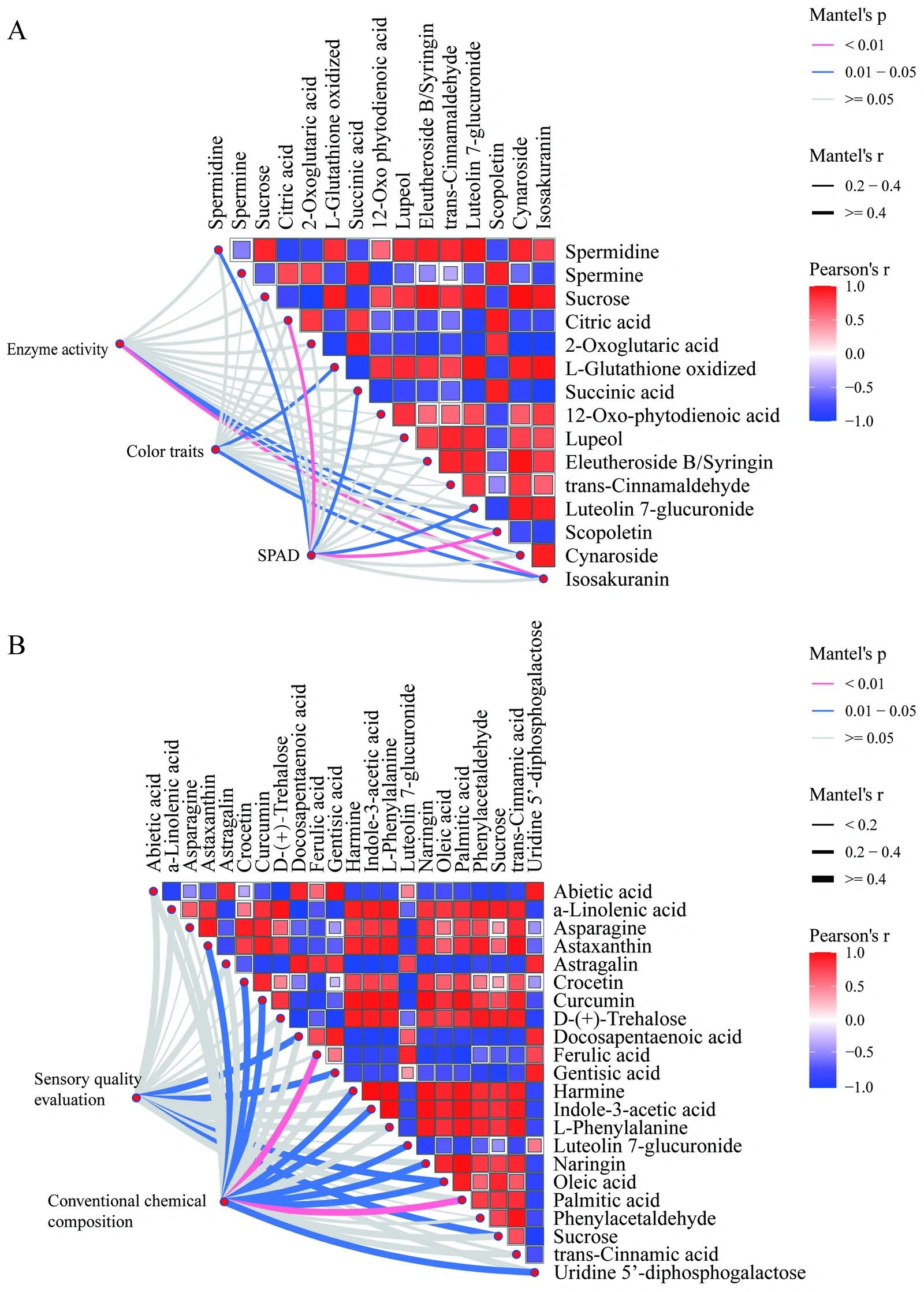
Correlation analysis between differential metabolites and physiological or biochemical traits. **(A)** Correlations between differential metabolites in fresh leaves and color traits, SPAD values, and enzyme activities. **(B)** Correlations between differential metabolites in flue-cured leaves and conventional chemical constituents and auxiliary flue-cured leaf trait indices. Correlations were calculated using Pearson’s correlation coefficient.

In flue-cured leaves, oleic acid, palmitic acid, phenylacetaldehyde, sucrose, and trans-cinnamic acid were mainly positively associated with conventional chemical constituents and auxiliary flue-cured leaf trait indices, whereas several other differential metabolites showed negative associations. These correlation patterns suggested that maturity-dependent changes in sugar metabolism, phenylalanine-derived metabolism, and lipid-associated metabolism were jointly associated with the biochemical trait profile of flue-cured leaves.

Based on the integrated results of phenotype, enzyme activities, untargeted metabolomics, and correlation analysis, we proposed a data-supported working hypothesis for maturity-associated metabolic remodeling in N. tabacum leaves (**Figure 8**). With increasing harvest maturity, fresh leaves showed progressive chlorophyll degradation and yellowing, differences in sucrose-related enzyme activities, reduced abundance of several TCA-cycle intermediates, and changes in metabolites assigned to phenylpropanoid- and redox-related pathways. After standard flue-curing, the maturity-associated metabolic profile was further characterized by changes in phenylalanine-derived metabolites, sugar- and amino-acid-related substrates, and lipid-associated metabolites. These coordinated associations provide a basis for the working hypothesis that increasing harvest maturity is linked to differences in fresh-leaf carbon allocation and in endpoint biochemical changes observed after flue-curing.

**Figure 8:**
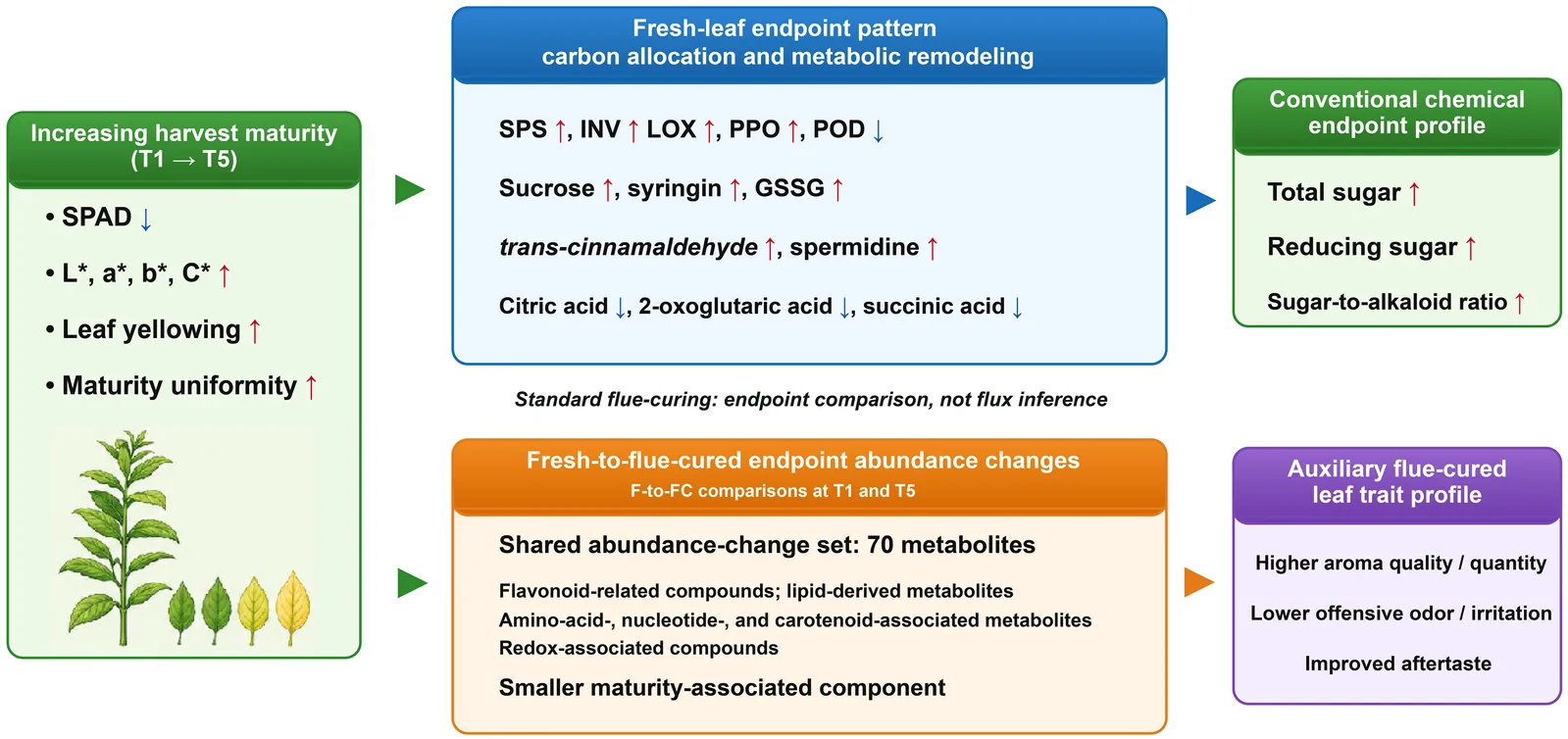
Working hypothesis for harvest-maturity-associated carbon allocation and fresh-to-flue-cured metabolite abundance changes in *Nicotiana tabacum* leaves. Increasing harvest maturity from T1 to T5 was associated with progressive leaf yellowing, reduced SPAD values, greater within-leaf maturity uniformity, differences in sucrose-related and oxidative enzyme activities, and changes in metabolites assigned to central-carbon-, phenylpropanoid-, and redox-related pathways. After flue-curing, harvest-maturity-associated differences were mainly reflected in phenylalanine-derived metabolites, sugar- and amino-acid-related substrates, and lipid-associated metabolites. The arrows summarize data-supported associations among these endpoint patterns and should not be interpreted as evidence of direct causal regulation, *de novo* metabolite biosynthesis, or confirmed metabolic flux. The pathway-level organization of representative differential metabolites is shown in **Figure 5**.

## 4 Discussion

### 4.1 Leaf maturation is accompanied by coordinated pigment degradation and developmental synchronization

The present study used harvest maturity level as a developmental gradient to investigate maturity-associated metabolic reprogramming in *N*. *tabacum* leaves. Increasing maturity from T1 to T5 was accompanied by progressive yellowing, a marked decrease in SPAD values, and significant increases in L*, a*, b*, and C*, indicating that the leaves gradually entered a more advanced maturation state. Leaf color changes in tobacco are closely associated with chlorophyll and carotenoid degradation, water status, and polyphenol-related processes (Fan et al., 2026; Meng et al., 2024; Wu et al., 2020). Therefore, the observed phenotypic changes should not be interpreted only as external maturity indicators, but also as visible manifestations of internal pigment turnover and metabolic transition. This interpretation is consistent with the view that leaf maturation and senescence involve coordinated chloroplast dismantling, carbon–nitrogen reallocation, and nutrient remobilization rather than only visible pigment loss (Guo et al., 2021; Havé et al., 2017).

The convergence of SPAD values among the leaf tip, middle, and base from T3 onward suggested that increasing harvest maturity improved within-leaf developmental uniformity. This is biologically meaningful because heterogeneous maturation within a single leaf may result in uneven substrate distribution and inconsistent postharvest biochemical transformation. In this study, T5 leaves showed the most advanced maturity phenotype, together with higher soluble sugar accumulation and more coordinated chemical composition after flue-curing. Similar links among leaf age, dehydration behavior, carbohydrate turnover, and transformed-leaf chemical properties have been reported in tobacco (Chen et al., 2019a; Yamaguchi et al., 2013). These results indicate that higher harvest maturity improved developmental synchronization and provided a more uniform physiological background for subsequent biochemical conversion.

The regression analyses summarized in **Supplementary Table S3** support a continuous but trait-specific physiological and conventional chemical maturity gradient across T1–T5. However, because untargeted metabolomics was performed only for T1 and T5, these results cannot establish a corresponding five-stage metabolomic trajectory. The metabolomic data should therefore be interpreted as endpoint contrasts between the lower- and higher-maturity treatments. The intermediate metabolomic states of T2–T4 remain unresolved, and future studies including intermediate stages, particularly T3, will be needed to determine whether individual metabolites change linearly, nonlinearly, transiently, or mainly at later harvest-maturity stages.

T5 should therefore be interpreted as the higher-maturity endpoint examined under the present experimental conditions, rather than as a universally optimal harvest stage. Defining an appropriate harvest window will require intermediate-stage metabolomic profiling, integrated agronomic and quality evaluation, and validation across additional environments and production years.

### 4.2 Maturity-associated carbon reallocation is reflected by sugar metabolism and TCA-cycle intermediates

Carbon allocation was one of the central processes associated with harvest maturity in this study. SPS and INV activities increased significantly in T5 fresh leaves, consistent with the higher total sugar and reducing sugar contents detected after flue-curing. SPS is a key enzyme involved in leaf sucrose synthesis and carbon partitioning, whereas INV participates in sucrose cleavage and hexose formation (Cheng et al., 2023; Huber and Huber, 1992). The higher SPS and INV activities at T5 were consistent with maturity-associated changes in sucrose synthesis- and cleavage-related processes. However, because transcript abundance and metabolic flux were not measured, these activity differences should not be interpreted as direct evidence that harvest maturity increased pathway flux.

At the metabolite level, citric acid, 2-oxoglutaric acid, and succinic acid were lower in T5 fresh leaves, indicating that higher harvest maturity altered the status of central carbon metabolism. The plant TCA cycle functions not only in respiratory energy production but also as a metabolic hub supplying carbon skeletons for biosynthetic processes and carbon-nitrogen interactions (Sweetlove et al., 2010). Therefore, the decline in these intermediates is more appropriately interpreted as maturity-dependent metabolic rebalancing rather than a simple reduction in metabolic activity. In particular, 2-oxoglutaric acid is closely connected with amino acid metabolism and 2-oxoglutarate-dependent dioxygenase reactions involved in specialized metabolite biosynthesis, including flavonoid-related pathways (Farrow and Facchini, 2014). Together with changes in sugar-related enzyme activities, these results suggest maturity-associated differences in the balance among carbon supply, respiratory metabolism, and downstream secondary metabolism.

In fresh leaves, this carbon rebalancing may support the transition from active photosynthetic growth to maturation. The altered availability of sugars, organic acids, and amino acid-derived substrates may also influence dehydration-associated biochemical reactions during subsequent flue-curing. Amino acid-sugar reactions and Maillard-type reactions have long been recognized as important biochemical processes during flue-curing (Banožić et al., 2020; Dickerson et al., 1976). Thus, maturity-dependent carbon reallocation provides an important metabolic connection between leaf developmental status and subsequent biochemical conversion.

### 4.3 Phenylpropanoid and redox-associated pathways are major components of secondary metabolic remodeling

Fresh T5 leaves showed higher relative abundances of syringin and trans-cinnamaldehyde, indicating enhanced accumulation of compounds associated with phenylpropanoid metabolism. Phenylpropanoid metabolism contributes to lignin biosynthesis, phenolic accumulation, antioxidant regulation, cell-wall modification, and the formation of volatile or bound aroma precursors (Liu et al., 2022; Wang et al., 2025). Previous studies have shown that aroma precursor biosynthesis and accumulation vary across tobacco growth stages (Liu et al., 2022), and changes in phenylpropanoid-related compounds are often associated with maturity-dependent leaf biochemical properties. In the present study, the enrichment of phenylpropanoid biosynthesis and the higher relative abundances of syringin and trans-cinnamaldehyde were associated with the higher-maturity stage.

Redox-related metabolism was also strongly involved in maturity-associated remodeling. T5 fresh leaves accumulated oxidized glutathione, indicating a shift in cellular redox status. Meanwhile, LOX and PPO activities increased, whereas POD activity decreased. These enzyme-activity and metabolite differences were consistent with an altered oxidative state at the higher-maturity stage. Such redox-associated differences may coincide with chlorophyll degradation, membrane lipid remodeling, phenolic oxidation, and subsequent postharvest biochemical transformation. The increase in spermidine further supports the involvement of polyamine metabolism in developmental transition and redox regulation. Polyamines are closely related to membrane stability, antioxidant capacity, and stress adaptation, and their accumulation may be associated with the physiological transition from green leaves to mature leaves.

Together, the changes in phenylpropanoid-related compounds, glutathione metabolism, polyamine metabolism, and oxidative enzymes indicate that higher harvest maturity is not only a process of pigment loss and carbohydrate conversion, but also a coordinated secondary metabolic transition. These associated changes may reflect differences in antioxidant status, phenolic metabolism, and substrate availability before subsequent flue-curing; their functional roles require further validation.

The enrichment pattern indicates that the fresh leaf response should be interpreted as a broad remodeling of plant secondary metabolism rather than as a single-pathway response. The enrichment of plant secondary metabolite biosynthesis, plant hormone biosynthesis, terpenoid/steroid biosynthesis, and phenylpropanoid-related pathways, together with changes in sucrose, polyamines, oxidized glutathione, and central-carbon-related metabolites, supports the view that higher harvest maturity was accompanied by coordinated differences in carbon-associated, hormone- or oxylipin-related, secondary-metabolic, and redox-related traits before flue-curing.

### 4.4 Fresh-to-flue-cured abundance differences involve aromatic amino acid-, transporter-associated substrate-, and lipid-related metabolite groups

Flue-curing is a postharvest process involving dehydration, residual enzymatic activity, heat-associated reactions, enzyme inactivation, pigment degradation, Maillard-type reactions, and broad changes in metabolite composition and detectability (Banožić et al., 2020; Chen et al., 2019a; Wang et al., 2025). Therefore, differences between fresh and flue-cured leaves in this study should be interpreted as endpoint differences in relative metabolite abundance rather than as direct evidence of *de novo* biosynthesis or a single biochemical conversion mechanism.

In the present within-site harvest-maturity design, the flue-cured endpoint comparison FC_T5 vs. FC_T1 involved aromatic amino-acid-related metabolite groups, especially phenylalanine- and tryptophan-associated metabolites, together with secondary metabolism, plant hormone-related categories, and transporter-associated substrate metabolites. Several putatively annotated phenylalanine-derived metabolites showed lower relative abundance in FC_T5 than in FC_T1, suggesting harvest-maturity-associated differences in the abundance of aromatic and phenolic-related metabolites after flue-curing.

In this context, the enrichment of phenylalanine- and tryptophan-related categories suggests that aromatic amino-acid-associated metabolite groups contributed to the endpoint differences observed after flue-curing (Cai et al., 2026; Wang et al., 2025), but these annotations should not be interpreted as direct evidence of pathway flux or hormone-related regulation.

The enrichment of ABC transporter-related KEGG terms indicates that metabolites assigned to transporter-associated substrate categories differed between FC_T5 and FC_T1. Because transcript, protein, or targeted transport assays were not performed, this result was interpreted as evidence of altered transporter-associated substrate patterns rather than direct evidence of transporter expression, transporter activity, or compartmental redistribution (Wu et al., 2020, 2026).

The direct fresh-to-flue-cured comparisons provided an analytical layer for distinguishing harvest-maturity-associated differences from fresh-to-flue-cured endpoint abundance changes. The large numbers of differential metabolites in FC_T1 vs. F_T1 and FC_T5 vs. F_T5 indicate that the metabolite profiles differed strongly between fresh and flue-cured tissues at both harvest-maturity endpoints (Meng et al., 2022; Wei et al., 2024; Wu et al., 2026). The 70 shared metabolites between these two comparisons suggest a common set of fresh-to-flue-cured abundance changes involving flavonoid-related compounds, fatty-acid-derived metabolites, amino-acid-related substrates, nucleotide-associated metabolites, carotenoid-related compounds, and redox-associated metabolites (Cai et al., 2026; Li et al., 2026). These endpoint differences are consistent with the biochemical complexity of flue-curing, during which dehydration, residual enzyme activity, oxidation, thermal reactions, non-enzymatic reactions, volatilization, and changes in metabolite extractability may jointly influence the detected metabolite pool (Li et al., 2025; Wang et al., 2025; Xu et al., 2025).

The direct fresh-to-flue-cured comparisons further suggested that endpoint abundance differences were not completely independent of harvest maturity. The lower- and higher-maturity endpoint comparisons contained distinct comparison-specific metabolites, indicating that the fresh-leaf biochemical starting point may influence the endpoint metabolite profile observed after flue-curing (**Figure 9**; **Supplementary Table S1**). This analytical framework builds on previous tobacco metabolomic studies showing that harvest maturity and curing conditions are associated with cured-leaf metabolite profiles, while further emphasizing paired fresh-to-flue-cured endpoint comparisons within a single-site maturity gradient (Cai et al., 2026; Wu et al., 2026). Together, these results support a working hypothesis in which harvest maturity is associated with fresh-leaf carbon allocation, redox status, and secondary metabolism, and these differences are reflected in shared and maturity-specific endpoint abundance patterns after flue-curing.

**Figure 9:**
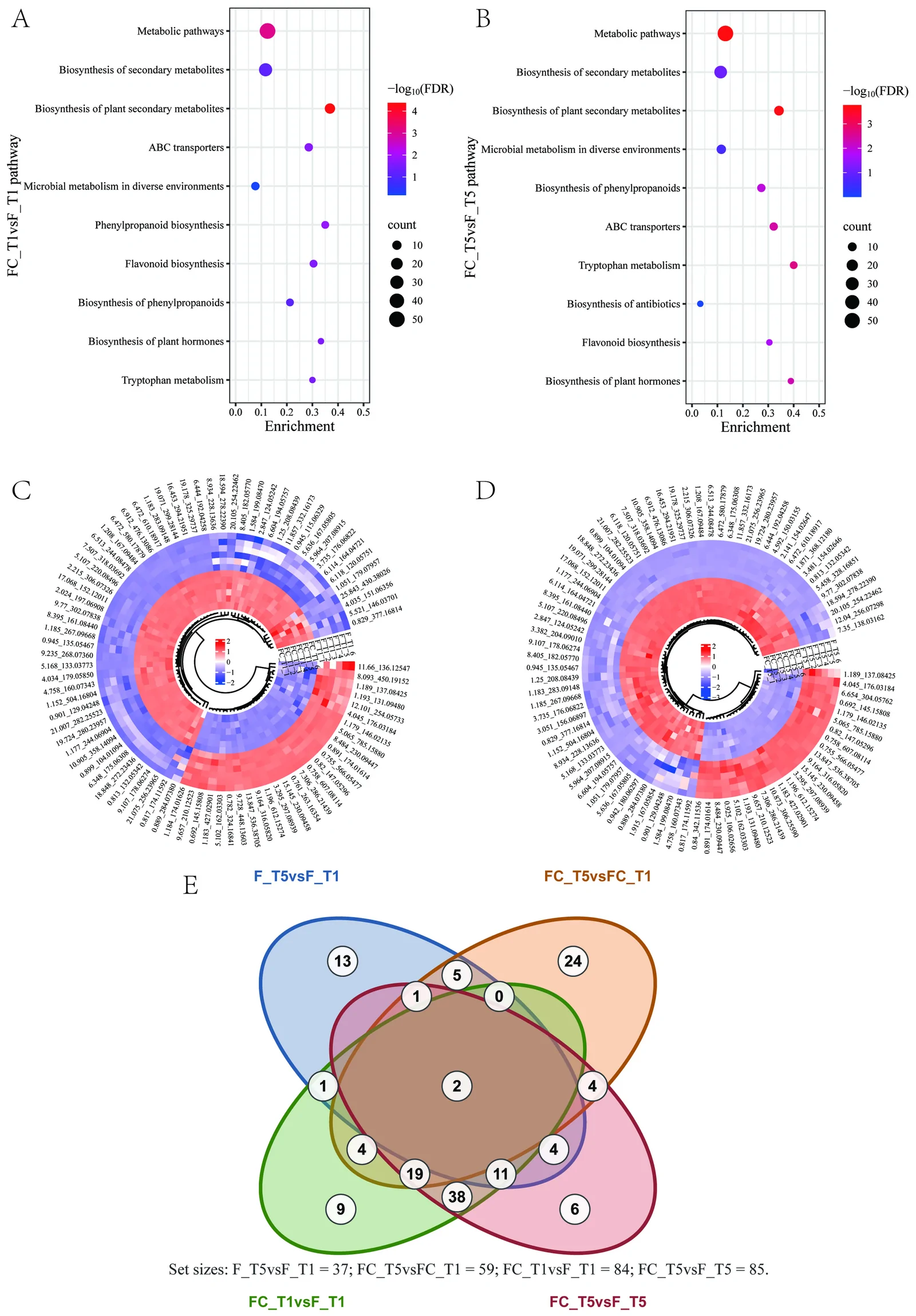
Direct fresh-to-flue-cured comparisons and overlap analysis of differential metabolites. **(A)** KEGG enrichment analysis of differential metabolites in the FC_T1 vs. F_T1 comparison. **(B)** KEGG enrichment analysis of differential metabolites in the FC_T5 vs. F_T5 comparison. **(C)** Circular heatmap showing differential metabolite abundance patterns in the FC_T1 vs. F_T1 comparison. **(D)** Circular heatmap showing differential metabolite abundance patterns in the FC_T5 vs. F_T5 comparison. **(E)** Venn diagram showing the overlap of differential metabolites among F_T5 vs. F_T1, FC_T5 vs. FC_T1, FC_T1 vs. F_T1, and FC_T5 vs. F_T5. The FC_T1 vs. F_T1 and FC_T5 vs. F_T5 comparisons identified 84 and 85 differential metabolites, respectively, and shared 70 metabolites, indicating a broad shared set of fresh-to-flue-cured metabolite abundance changes together with harvest-maturity-associated, comparison-specific components. Broad KEGG categories, such as protein digestion and absorption, are database-derived annotation categories and were not interpreted as direct biological processes occurring in tobacco leaves. Metabolite ID-to-name mapping for panels C and D is provided in **Supplementary Table S2**.

Previous studies on postharvest browning and maturity-related aroma glycosides also indicate that phenylalanine- and phenylpropanoid-related metabolites are dynamically transformed during leaf maturation and postharvest conversion (Zhao et al., 2023a; He et al., 2015). In the present study, the decreases in phenylacetaldehyde, trans-cinnamic acid, and related metabolites in the endpoint comparisons support an association between harvest maturity and both metabolite accumulation in fresh leaves and subsequent metabolite abundance changes after harvest.

The decreases in sucrose and several amino acid-related metabolites after flue-curing may reflect enhanced substrate utilization in leaves at higher maturity. Carbohydrates and amino acid-derived compounds participate in dehydration-associated biochemical reactions, carbon–nitrogen conversion, and Maillard-type reactions during flue-curing (Dickerson et al., 1976; Banožić et al., 2020), thereby contributing to the postharvest biochemical profile of flue-cured leaves. Curing-related metabolite transformation and enzymatic browning may further reshape sugar-, amino acid-, and phenolic-derived substrates (Wei et al., 2024; Chen et al., 2019b). Together, these changes suggest that higher harvest maturity was associated with differences in the substrate pool available for biochemical changes during flue-curing.

Lipid-associated metabolites were also involved in the maturity-dependent postharvest response. Changes in fatty acids such as oleic acid, palmitic acid, linoleic acid, and related lipid-derived metabolites may reflect membrane lipid remodeling, lipid oxidation, or altered availability of lipid-derived substrates during flue-curing. Although lipid metabolism was not the only enriched module in the updated KEGG analysis, fatty-acid-related metabolites were repeatedly detected across maturity and fresh-to-flue-cured comparisons. These results suggest that lipid-associated remodeling should be interpreted together with aromatic amino acid metabolism, secondary metabolism, transporter-associated substrate patterns, and carbohydrate-related substrate conversion when evaluating maturity-associated postharvest metabolic patterns (**Figure 5**).

### 4.5 Candidate metabolites link developmental maturity with physiological and biochemical traits

Correlation analysis was used to examine associations between developmental physiology and metabolite accumulation. In fresh leaves, sucrose, citric acid, and succinic acid were positively associated with SPAD and/or color-related parameters, whereas oxidized glutathione showed the opposite pattern. Spermidine was mainly linked with maturity-related physiological traits and oxidative enzyme activities, suggesting the involvement of polyamine metabolism in the maturity-associated redox adjustment. These compounds may serve as candidate metabolic indicators of leaf maturity and pre-flue-curing biochemical state. In particular, the correlations involving sucrose and organic acids support the role of carbon allocation in maturity progression (Igamberdiev and Eprintsev, 2016), while the association of oxidized glutathione and spermidine with physiological traits suggests the involvement of redox regulation and polyamine metabolism (Chen et al., 2019).

In flue-cured leaves, oleic acid, palmitic acid, phenylacetaldehyde, sucrose, and trans-cinnamic acid were mainly associated with conventional chemical constituents and auxiliary postharvest trait indices. Previous integrative metabolomic studies in tobacco have shown that metabolite profiles can be linked with postharvest leaf traits (Zhao et al., 2023b). In the present study, the correlation results indicate associations between the postharvest biochemical trait profile and maturity-dependent differences in sugar-, phenylalanine-derived-, and lipid-associated metabolites. These metabolites may therefore serve as candidate indicators linking preharvest maturity with postharvest biochemical outcomes.

These relationships remain correlative. Although the integrated physiological, enzyme-activity, metabolomic, and correlation data support the working hypothesis summarized in **Figure 8**, they do not establish causal links among harvest maturity, pathway activity, metabolite flux, and postharvest biochemical traits. Targeted metabolite quantification, gene-expression analysis, isotope tracing, and time-course curing experiments will be needed to validate these proposed relationships.

Overall, the results support a working hypothesis in which increasing harvest maturity is associated with coordinated differences in central-carbon-related metabolites, phenylpropanoid accumulation, redox status, and substrates involved in fresh-to-flue-cured endpoint abundance changes. The direct fresh-to-flue-cured comparisons further indicate a broad shared abundance-change component and a smaller harvest-maturity-associated component. These findings identify harvest maturity as an important factor associated with both fresh-leaf biochemical status and the endpoint metabolite abundance profile observed after flue-curing. Future studies combining targeted metabolite quantification, volatile analysis, gene expression analysis, isotope tracing, and time-course sampling during flue-curing would help clarify the dynamic conversion of individual metabolites and improve mechanistic understanding of postharvest biochemical traits.

## 5 Conclusions

Increased harvest maturity in *N. tabacum* leaves was associated with progressive yellowing, decreased SPAD values, altered sucrose-related enzyme activities, and broad endpoint metabolomic differences. The T1–T5 treatments formed a continuous but trait-specific physiological and conventional chemical maturity gradient, but untargeted metabolomics was limited to the contrasting T1 and T5 endpoints. Thus, the metabolomic results should be interpreted as endpoint contrasts rather than as a complete five-stage metabolomic trajectory. The higher-maturity endpoint was associated with differences in carbon-associated, secondary-metabolic, phenylpropanoid-related, aromatic amino-acid-related, transporter-associated, and lipid-associated metabolite groups. Direct fresh-to-flue-cured comparisons further indicated both shared metabolite abundance changes and harvest-maturity-associated, comparison-specific components. These findings suggest that harvest maturity is associated with both the fresh-leaf biochemical starting point and the endpoint metabolite profile after flue-curing, while causal mechanisms require further targeted and time-course validation.

## Data Availability

The data supporting this study are available in Mendeley Data at https://doi.org/10.17632/9fbv5cgf4p.1. Additional data are available from the corresponding authors upon reasonable request.

## Supplementary material

Supplementary Figure 1 Classification of identified metabolites and pathway-category distribution of differential metabolites across comparison groups.

Supplementary Figure 2 PCA score plot showing QC sample clustering and overall metabolomic sample distribution.

Supplementary Figure 3 Two-factor analysis of metabolite abundance using harvest maturity, sample state, and maturity × sample-state interaction.

Supplementary Table 1 Identified and differential metabolites, including annotation confidence levels.

Supplementary Table 2 Metabolite ID-to-name mapping for circular heatmaps in **Figures 9C, D** and **Figure 6**.

Supplementary Table 3 Linear and quadratic trend analyses of physiological and biochemical traits across T1–T5 harvest-maturity treatments.

Supplementary Table 4 Two-factor analysis of metabolite abundance using harvest maturity, sample state, and maturity × sample-state interaction.
