# Supplementary material for: Harvest maturity is associated with carbon reallocation and fresh-to-flue-cured metabolic changes in Nicotiana tabacum leaves
Source: Front Plant Sci. 2026 Sep 21;17:1939922. doi: 10.3389/fpls.2026.1939922 (PMC13635354; doi:10.3389/fpls.2026.1939922)
Supplement: Supplementary file 1 [file Image2.pdf]

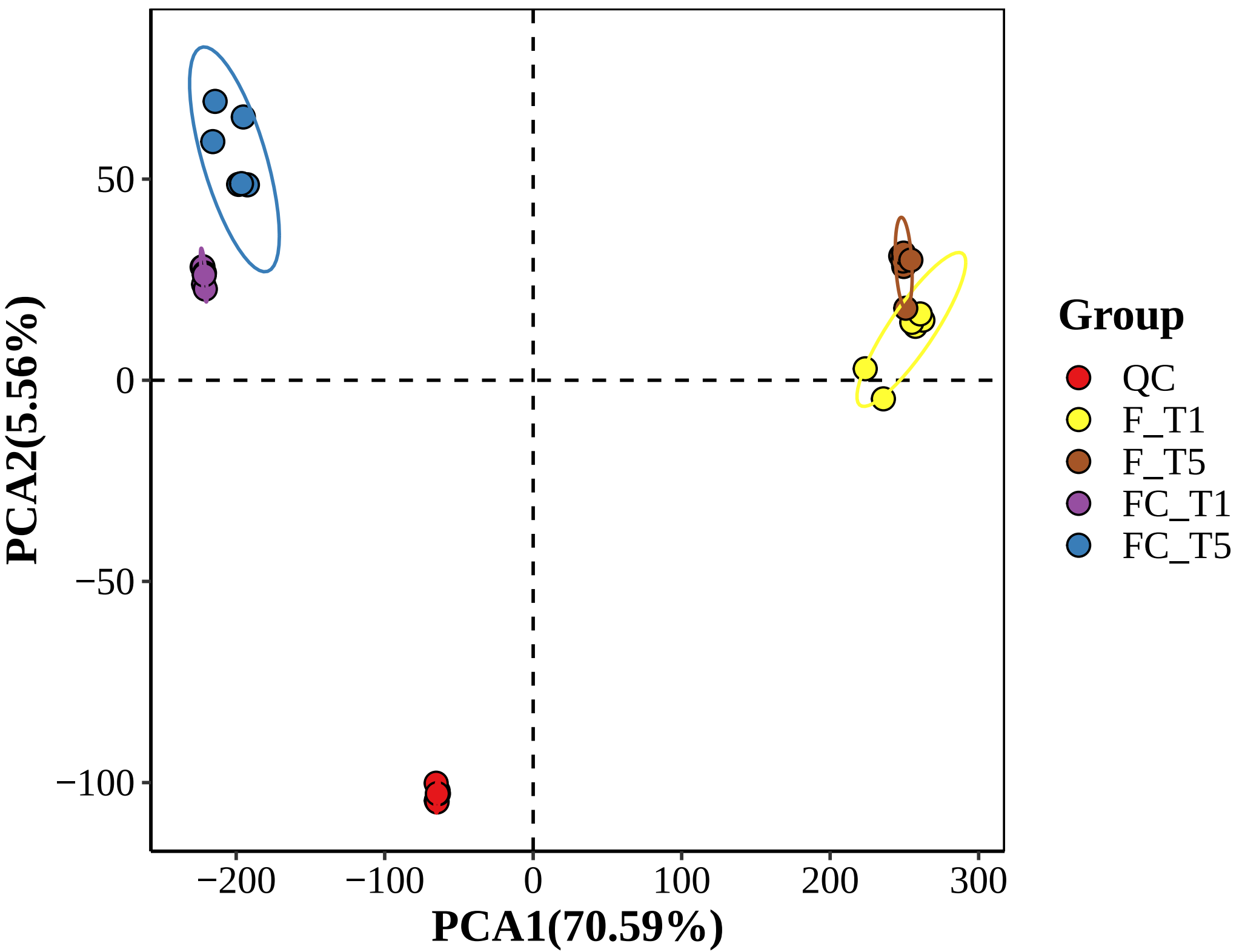

PCA2(5.56%)
PCA1(70.59%)
50
0
−50
−100
−200
−100
0
100
200
300
Group
QC
F_T1
F_T5
FC_T1
FC_T5
